# Event-related brain potentials reflect predictive coding of anticipated economic change

**DOI:** 10.3758/s13415-020-00813-5

**Published:** 2020-08-18

**Authors:** Diamantis Petropoulos Petalas, Stefan Bos, Paul Hendriks Vettehen, Hein T. van Schie

**Affiliations:** 1 Behavioural Science Institute, Faculty of Social Sciences, Radboud University, Nijmegen, Netherlands; 2 Department of Communication Science, Faculty of Social Sciences, Free University of Amsterdam, De Boelelaan 1105 (3E), 1081 HV Amsterdam, The Netherlands; 3 Department of Medical Biology, Faculty of Science, Radboud University, Nijmegen, Netherlands

**Keywords:** Attention, Cognitive control, Decision-making, ERP, Economic forecasting, Predictive coding

## Abstract

Research has demonstrated the importance of economic forecasts for financial decisions at the aggregate economic level. However, little is known about the psychological and neurophysiological mechanisms that economic forecasts activate at the level of individual decision-making. In the present study, we used event-related brain potentials (ERPs) to test the hypothesis that economic forecasts influence individuals’ internal model of the economy and their subsequent decision behavior. Using a simple economic decision-making game, the Balloon Analogue of Risk Task (BART) and predictive messages about possible economic changes in the game before each block, we test the idea that brain potentials time-locked to decision outcomes can vary as a function of exposure to economic forecasts. Behavioural results indicate that economic forecasts influenced the amount of risk that participants were willing to take. Analyses of brain potentials indicated parametric increases of the N1, P2, P3a, and P3b amplitudes as a function of the level of risk in subsequent inflation steps in the BART. Mismatches between economic forecasts and decision outcomes in the BART (i.e., reward prediction errors) were reflected in the amplitude of the P2, P3a, and P3b, suggesting increased attentional processing of unexpected outcomes. These electrophysiological results corroborate the idea that economic messages may indeed influence people’s beliefs about the economy and bias their subsequent financial decision-making. Our findings present a first important step in the development of a low-level neurophysiological model that may help to explain the self-fulfilling prophecy effect of economic news in the larger economy.

**Electronic supplementary material:**

The online version of this article (10.3758/s13415-020-00813-5) contains supplementary material, which is available to authorized users.

Economic forecasting refers to the psychology of making predictions about the future economy. Economic forecasts can have important implications for people’s economic expectations and everyday economic behaviour. For instance, the anticipation of an economic opportunity or threat may lead individuals to opt for riskier strategies to increase financial profit or to choose safer options with lower payoffs to secure existing resources. Research on economic forecasting is concerned with the effect of expectations on economic decision-making and self-fulfilling prophecies (i.e., economic consequences) that may follow from these expectations (Bovi, 2009; Diekmann, Tenbrunsel, & Galinsky, 2003; Greenwood & Shleifer, 2014; Petropoulos Petalas, van Schie, & Hendriks Vettehen, 2017; Wennberg & Nykvist, 2007). The field of economic forecasting is part of a larger literature on decision-making psychology that focusses on probabilistic forecasting in various domains, such as economy, weather, and health (Brown, 1973; Christensen-Szalanski & Bushyhead, 1981; Lawrence, Goodwin, O’Connor, & Önkal, 2006).

Economic forecasts are often represented in media news (Bach, Weber, & Quiring, 2013; Doms & Morin, 2004; Goidel, Procopio, Terrell, & Wu, 2010; Kalogeropoulos, Albæk, de Vreese, & Van Dalen, 2015; Tetlock & Gardner, 2015). Therefore, “effects” of economic forecasts on decision-making behaviour are often discussed in studies on economic/financial news that investigate formation of economic perceptions or beliefs and how they link to collective financial decision-making phenomena at different levels of aggregate economic behaviour (Arts, Takeshita, & Becker, 2002; Boomgaarden, van Spanje, Vliegenthart, & de Vreese, 2011; Giglio & Shue, 2014; Hetsroni, Sheaffer, Ben Zion, & Rosenboim, 2012; Lischka, 2015; Pruitt, Reilly, & Hoffer, 1988; van Raaij, 1989). Examples of such decision-making phenomena span from stock market reactions and investment patterns (Engelberg & Parsons, 2011; Green, 2004; Hetsroni, Reizer, & Ben Zion, 2017; Roache & Rossi, 2010; Scheufele, Haas, & Brosius, 2011; Tetlock, 2007) to consumer and household spending (Bovi, 2009; Doms & Morin, 2004; Kamins, Folkes, & Perner, 1997; Starr, 2012).

However, and despite the apparent implication of economic forecasts for economic behaviour, we know relatively little about how economic forecasts set in in the minds of individuals and how they influence financial decision-making that may lead to such collective behavioural phenomena. Characterizing these mechanisms can be useful in the modeling and understanding of real-world economic developments, as well as for predicting or even controlling such developments to prevent or counteract the negative effects of bad investments and recurring economic crises.

## Towards a cognitive psychological account of economic forecasting

Frydman and Camerer (2016) reviewed psychological and neuroscientific literature to discuss psychophysiological mechanisms underlying biases in economic decision-making. Such biases can be driven by domain-general cognitive operations supporting perception, action, memory, and motivation (Frydman & Nave, 2015). For example, negative emotional information is processed more attentively and remembered better than positive information (Compton, 2003). Similarly, investors have been found to react asymmetrically to positive and negative economic forecasts (Kuhnen, 2015; Soroka, 2006), and specifically more strongly to negative than to positive economic news (Akhtar, Faff, Oliver, & Subrahmanyam, 2011).

Another theoretical framework in cognitive neuroscience that may be relevant to understand the influence of economic forecasting in financial decision-making is the *predictive processing framework* (Clark, 2013; Den Ouden, Kok, & de Lange, 2012; Friston, 2005, 2010; Hohwy, 2013; Huang & Rao, 2011; Rao & Ballard, 1999; Rauss, Schwartz, & Pourtois, 2011; Rushworth, Mars, & Summerfield, 2009; Summerfield et al., 2006; Summerfield & de Lange, 2014). In its core idea, the brain is considered a hierarchical prediction-generation machinery that constructs and holds an internal model of the environment, by favoring higher-level beliefs (priors) that match lower-level sensory input at multiples levels of the processing hierarchy (i.e., a process of reconciling sensory information with existing mental schemata). By transmitting prediction error signals (i.e., neural responses to mismatches between prior beliefs and new sensory input) to higher processing units, representations of the world can be neuronally updated in a highly efficient manner (Hsu, Hämäläinen, & Waszak, 2014; Huang & Rao, 2011). Although predictive processing originated in visual research, prediction error signals have been identified and discussed in relation to economic decision-making as well (Frydman & Camerer, 2016; Knutson, Wimmer, Kuhnen, & Wilkielman, 2008; Kuhnen & Knutson, 2005; Preuschoff, Bossaerts, & Quartz, 2006; Rushworth et al., 2009; Summerfield & Tsetsos, 2012; Towal, Mormann, & Koch, 2013). For instance, distinct neural circuits, including the nucleus accumbens in the ventral striatum and the anterior insula, have been found to index reward anticipation and reward prediction errors. Overactivation and underactivation in these respective regions may lead to shifts in risk preferences and promote irrational choice during risky decision-making (Engelmann, Meyer, Fehr, & Ruff, 2015; Kuhnen & Knutson, 2011).

In line with the predictive processing framework, Petropoulos Petalas et al. (2017) recently proposed that economic forecasts may directly influence people’s prior beliefs about the economy and their perception of future economic events and financial decision options. In their experiment, participants played a gambling task (the Balloon Analogue Risk Task, or BART), and while playing, they received a message informing them of possible changes in the BART payoff scheme that might occur in upcoming trials. The message either indicated a possible negative change in the BART, whereby balloons might be popping more easily, or the message indicated a possible positive change, whereby balloons might be popping at a later time. Results indicated that messages forecasting a negative development resulted in participants taking less risk than messages forecasting a positive development. In addition, a gradual increase in response time with each inflation step was found to be stronger following negative economic forecasting than following positive economic forecasting. Both effects were interpreted to be consistent with the theoretical idea that economic forecast may change people’s prior belief of the economy and influence their perception of risk in financial decision-making.

Here, we build on this paradigm to scrutinize the hypothesis that economic forecasting can influence people’s mental model of the economy and influence their anticipation of economic outcomes. More specifically, we will investigate prediction error signals reflected in the EEG that provide an implicit measure of participants anticipation of positive and negative economic outcomes in the BART. Previous research has identified several ERP components to display reward prediction errors, most notably the feedback related negativity (FRN) and the P3/P300 (Holroyd & Coles, 2002; Nieuwenhuis, Holroyd, Mol, & Coles, 2004; San Martín, 2012; Xu et al., 2016). The FRN represents a negative peak or negative difference wave over medial frontal regions between 200 milliseconds (hereafter, ms) and 400 ms following negative feedback or unexpected absence of reward (Nieuwenhuis et al., 2004; Walsh & Anderson, 2012). The P3 or P300 is a pronounced positive deflection occurring between 300 ms and 600 ms over the medial frontal and parietal and is known to be sensitive to the magnitude of the reward as well as violations of reward expectancy (San Martín, 2012; Yeung & Sanfey, 2004). As prediction errors may theoretically be expressed at various stages of outcome processing—from early visual processing as reflected in the N1 to slow waves following the P3 (Duncan-Johnson, 1981; West, Bailey, Anderson, & Kieffaber, 2014)—we took an exploratory approach to first identify components in the ERP that are sensitive to risk taking (inflation step) in the BART and subsequently tested whether and to what extent these components would be influenced by economic forecasting. The functional significance of the components identified and studied here is addressed extensively in the Discussion section.

## Method

In our study, the BART was framed as an economic decision-making computer game, in which participants could earn successively higher amounts of (actual) money by making riskier choices. The BART was chosen because it can be seen as an intuitively straightforward visual metaphor of the economy as a bubble at risk of a burst. The same metaphor of the bubble economy is often used in economic news and involves a common figure of speech (Shiller, 2015; van der Yeught, 2007) linking BART’s bubble economy with the concept of actual economic bubbles in the real world. Furthermore, the features of the BART resemble real-world risky decision-making in the sense that individuals often experience uncertainty about the outcomes of their financial decisions, as seen in market trading behaviour; investing in shares, bonds, or land property; starting up a new business; or buying a house. Likewise, the BART provides individuals with the option to avoid risk taking by collecting the value that was successfully accumulated up to that point, which may resemble risk aversive behaviour such as saving money in a zero-interest checking account.

While playing in the BART, participants encountered economic forecasting by means of a text message on the computer screen, which suggested possible changes (positive and negative) in the game’s economic state of future trials (as in Petropoulos Petalas et al., 2017). We recorded participants’ behavioural responses in the game, as well as their electroencephalogram (EEG). Our study used a within-subjects experimental design with three levels (blocks): a baseline and two experimental blocks. The baseline block was always presented first. Both experimental blocks were preceded by a positive or negative economic forecast (counterbalanced).

### Participants

We determined the sample size necessary for our experiment on the basis of a similar previous study that used 22 participants and reported moderate (*d* = .41 and η^2^ = .23) effect sizes (see S. Xu et al., 2016). To account for possible dropouts or errors during the experiment, 23 right-handed participants (nine males, *M*_age_ = 22.5 years, *SD*_age_ = 3.0 years; age range: 18–30 years), with no known visual or neurological impairments, were recruited for the experiment. Two female and one male participant had to be excluded due to errors in the correct registration of event markers in the raw EEG signal, and due to complications with electrode conductivity or impedance. All participants received a standard participation fee per time spent in the lab (€5.00 per hour). The 20 participants used in the final study sample received additional monetary profit ranging between €4.33 and €9.94 (*M* = 7.59, *SD* = ±1.54), based on 5% of their actual performance in the BART. All participants were recruited through the university’s participant repository portal and provided verbal and written consent. The study was conducted according to the ethical standards described in the 1964 Declaration of Helsinki.

### Paradigm

In the original BART (Lejuez et al., 2002), the participant can acquire an increasing monetary value by sequentially inflating a visual analogue of a balloon. At each trial, the participant is presented with the decision to either inflate the balloon or to withdraw from inflating it and collect the monetary value acquired to that point. Every successive inflation response leads to an increase in the size of the balloon and the monetary value associated with that particular balloon size, given that the inflation is successful. At the same time, each successive inflation response comes at an increasing probability for a burst. If the balloon bursts, any monetary value is lost. It is the participant’s choice to stop inflating the balloon when she or he feels the level of risk outweighs the possible gain. Therefore, each successive inflation response in the BART reflects risk taking, as it may result in either an increase in potential benefit (as the value of the balloon increases cumulatively with each response to inflate) or in a total loss of the balloon value accumulated up to that point. Following a balloon burst or a response to collect the acquired monetary value, the trial ends and the participant may start inflating a new balloon. In our modified version of the BART task, we used a visual analogue of a bubble, as it may be seen as a visual metaphor of a bubble economy at risk of a burst. Although the explicit meaning of this metaphor was not mentioned to participants, the word “bubble” instead of “balloon” was used throughout the experiment.

### BART task

Similar to the Petropoulos Petalas et al. (2017) study, here we used an adapted version of the BART paradigm with a maximum of 12 inflation steps that permits measurements of behavioural and electrophysiological data to successive inflation responses (self-paced). Participants were introduced to the BART as a game in which they could earn money depending on their performance and were specifically instructed that the game’s goal was to maximize their earnings. Although the exact probabilities for bursts were unknown to participants, they were told that each bubble could be maximally inflated 12 times before a definite burst would occur, and that they had to perform at least one inflation before collecting any money. That is, the first inflation response would never result in a burst. Bubble bursts were pseudorandomized and their probability of bursting was predetermined based on the following formula:

1 $$ {p}_{\mathrm{burst}}=\mathrm{Number}\ \mathrm{of}\ \mathrm{Inflation}\ \mathrm{Step}/12. $$

To ensure similar reward structure across the three blocks, the same sequence of burst probabilities was used in each block. The experiment was programmed in Presentation^®^ (Version 15.0, Neurobehavioural Systems Inc., Berkeley, CA, USA). Participants were instructed to use only their right (left) hand for inflating the bubble by pressing the space bar and their left (right) hand for collecting money using the left (right) control button (i.e., response hands for inflating and collecting money were counterbalanced across participants).

**Fig. 1:**
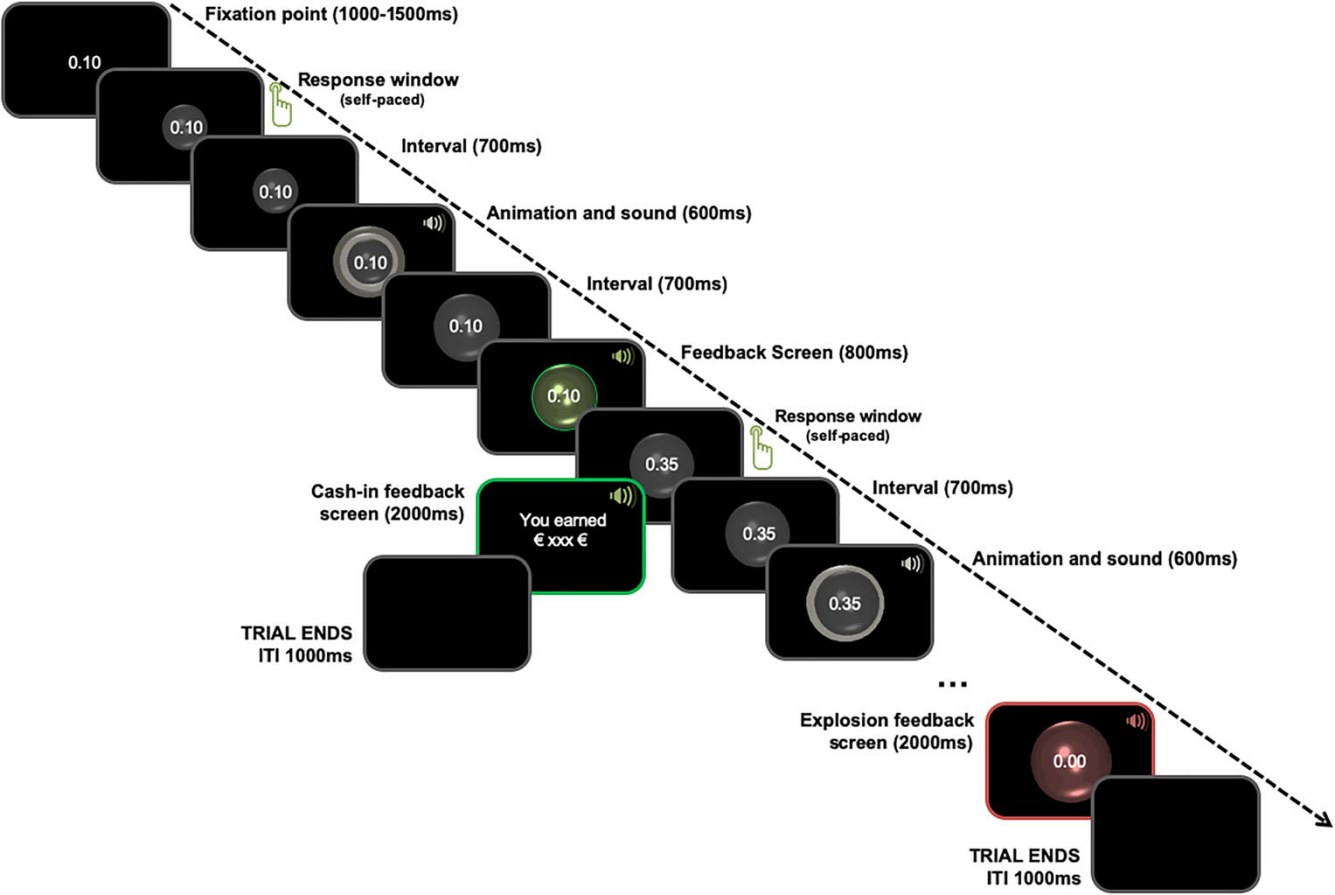
Time course of all events at a given trial of the BART task

At the beginning of each trial, the value “0.10” was presented at the center of the screen (font type: Arial; font size: 11 pt.; font colour: white), similar to a standard fixation point. Following a time interval that varied between 1,000 and 1,500 ms, a transparent, white-lined bubble, 100 × 100 pixels (1.33 × 1.33 inch [3.37 × 3.37 cm]), was projected around the presented value, and the participant could start to inflate the bubble (see Fig. 1). Inflation responses were self-paced. Seven hundred ms after participants pressed the space bar to inflate, the bubble began to increase in size (animation), and 300 ms later a short inflating sound (sound duration: 300 ms) was played; the total duration of this audiovisual frame was 600 ms. Each successive response to inflate the bubble led to an animated increase in its size by approximately 1^o^ visual angle (about 0.39 inch [1 cm] in all directions). Seven hundred ms after the animation frame, a positive or a negative feedback stimulus was presented, indicating whether the response resulted in a successful bubble inflation or in a bubble burst. In case of a successful inflation, the inflated bubble would turn green and remain on-screen for 800 ms while a short clinking sound (150 ms) was played (600 ms after the change in colour, to distinguish between visual and auditory evoked potentials). The bubble would then turn white again, and the value would update to the stake of the next inflation response. With each successive inflation step, the value increased, up to €3.90 (see Table S1 in the Supplementary Materials, which presents a detailed description of the bubble value at each successive Inflation Steps 1–13).

In case of a bubble burst, the inflated bubble would turn red and remain on-screen for 2 000 ms, while a subtle explosion sound (300 ms) was played (600 ms after the change in colour); no money would be collected in that case, and the trial would end. A collect response—that is, stop inflating the bubble and collect the accumulated value (using the right/left control button) would immediately result in a green-framed screen, with the text “*You collected* *value bubble* *Euro*” in the center of the screen for 2,000 ms (font type: Arial; font size: 22 pt.; font colour: green), and the same clinking sound was successively played for three times (600 ms after the change in colour). Following the end of every trial (i.e., after either a collect response or a bubble burst), a black screen was presented as an intertrial interval for 1,000 ms.

### Procedure

Participants were first welcomed in the lab and were introduced to the experimental equipment (EEG) and procedure. Following, participants provided their written consent, in accordance to the standard protocol for human psychology experimentation from the Ethics committee of the institute where the study was conducted. Next, participants were seated in a comfortable chair, approximately 80 cm in front of a 24-in. LCD monitor (TN panel type; 1,920 × 1,080 resolution; model BenqXL2420Z) and used a gaming keyboard (Corsair Vengeance K70) to perform the modified version of the BART task. After explaining the experimental procedure and fitting of the EEG electrodes (see Data Collection and EEG Signal Preprocessing section, below), participants received instructions about the BART task. Participants were familiarized to the BART with five practice trials (bubbles), ensuring they had understood how the game works and how to minimize artifacts in the EEG. Participants were explicitly instructed to use the intervals between successive rounds or trials in order to blink or move, when necessary. After completing this practice phase, a baseline block of 80 trials was presented in four mini blocks of 20 trials each. Following each block, a self-paced break of approximately 1 minute was offered to participants to relax before the next block. Following the baseline block, two experimental blocks were administered, again consisting of four mini blocks of 20 trials each. Before the onset of each experimental block, a positive or negative text message appeared on a black background screen (font type: Arial; font size: 28 pt.; font colour: green in the case of a positive message, red in the case of a negative message). The order of positive and negative forecasts before the experimental blocks was counterbalanced across participants. The positive (negative) forecast read: “Positive (negative) economic changes may occur within the coming trials. In this case, the chances of explosions will decrease (increase), which will result in bubbles popping at bigger (smaller) sizes, and can influence your total gains.” The forecasts were deliberately kept vague, in line with the theoretical proposition that economic news often offers a vague description of economic predictions about the future (Baker, Bloom, & Davis, 2016; Tetlock & Gardner, 2015). Therefore, the word “may” was used, which is a neutral phrase of estimative probability that says nothing about the degree of likelihood for and event to actually happen (Mauboussin & Mauboussin, 2018). In addition, the forecasts captured the element of directionality (i.e., valence), which is often accentuated or framed in journalistic reports about the future economy (Akhtar, et al., 2011; Soroka, 2006). Each message remained on-screen until participants chose to continue to the next block. Following the BART task, participants completed a short inventory of questions including items about demographics and items checking the message manipulation. The whole experiment lasted on average 100 minutes (*M* = 99.86, *SD* = 10.24), depending on participants’ pace.

### Data collection and EEG signal preprocessing

Behavioural performance data, including number of inflations performed per bubble (trial) and reaction times (RTs) in response to every inflation decision, were collected using the stimulus presentation software. EEG data were collected with two sets of 32-channel active electrode systems (actiCap MedCaT B.V., Netherlands), amplified by two 32-channel BrainAmp EEG amplifiers with electrode placement according to the international 10–20 system on the following scalp locations: Fp1/2, AFz, AF3/4, AF7/8, Fz, F1/2, F3/4, F5/6, F7/8, FCz, FC1/2, FC3/4, FC5/6, FT7/8, Cz, C1/2, C3/4, C5/6, CPz, CP1/2, CP3/4, CP5/6, TP7/8, Pz, P1/2, P3/4, P5/6, P7/8, POz, PO3/4, PO7/8, Oz, O1/2, PO10, and right mastoid. A ground electrode was placed on the right collarbone, and the reference electrode was placed at the left mastoid. Electrooculography (EOG) was recorded using electrodes at supra-orbital and infra-orbital sites around the right eye to control for vertical eye movement and blinks, and at the outer canthi of the left and right eyes to control for horizontal eye movements. Conductance gel was used to connect the electrodes to the scalp, and impedance was kept below 20 kΩ. The online sampling rate was set at 500 Hz.

Preprocessing of the EEG data was done in BrainVision Analyzer (Version 2.0.2, Brain Products GmbH, Gilching, Germany). The EEG signal was rereferenced off-line to an average of the linked mastoids. A Butterworth zero-phase filter was used to eliminate low-frequency drift and high-frequency noise, with a low cutoff set at 0.1 Hz at 48 dB/octave and a high cutoff at 40 Hz at 48 dB/octave. Eye-movement artifacts (blinks and saccades) were corrected using the Gratton and Coles ocular correction method (Gratton, Coles, & Donchin, 1983). Muscle artifacts and low-frequency drifts were excluded using semi-automatic detection of amplitudes surpassing ±150 μV. Manual detection was then used to exclude any remaining artifacts that were missed in the automatic detection procedure. On average, 7% of the recorded EEG signal was excluded from analysis.

The stimulus presentation script was programmed to send triggers to the EEG data acquisition system, marking all stimuli and responses in parallel with the recording of the EEG. Markers designating feedback stimuli (indicating either a successful or an unsuccessful inflation) were used to extract epochs between −250 ms and +750 ms with respect to the onset of feedback stimulus. Epochs were averaged separately per stimulus type (successful/unsuccessful inflation), per inflation step (Step 1–Step 12), per experimental condition (baseline, positive message, and negative message) and electrode site. These epochs were then baseline corrected (from −250 ms to 0 ms) to create ERPs relevant to our investigation.

#### EEG components of interest

**Fig. 2:**
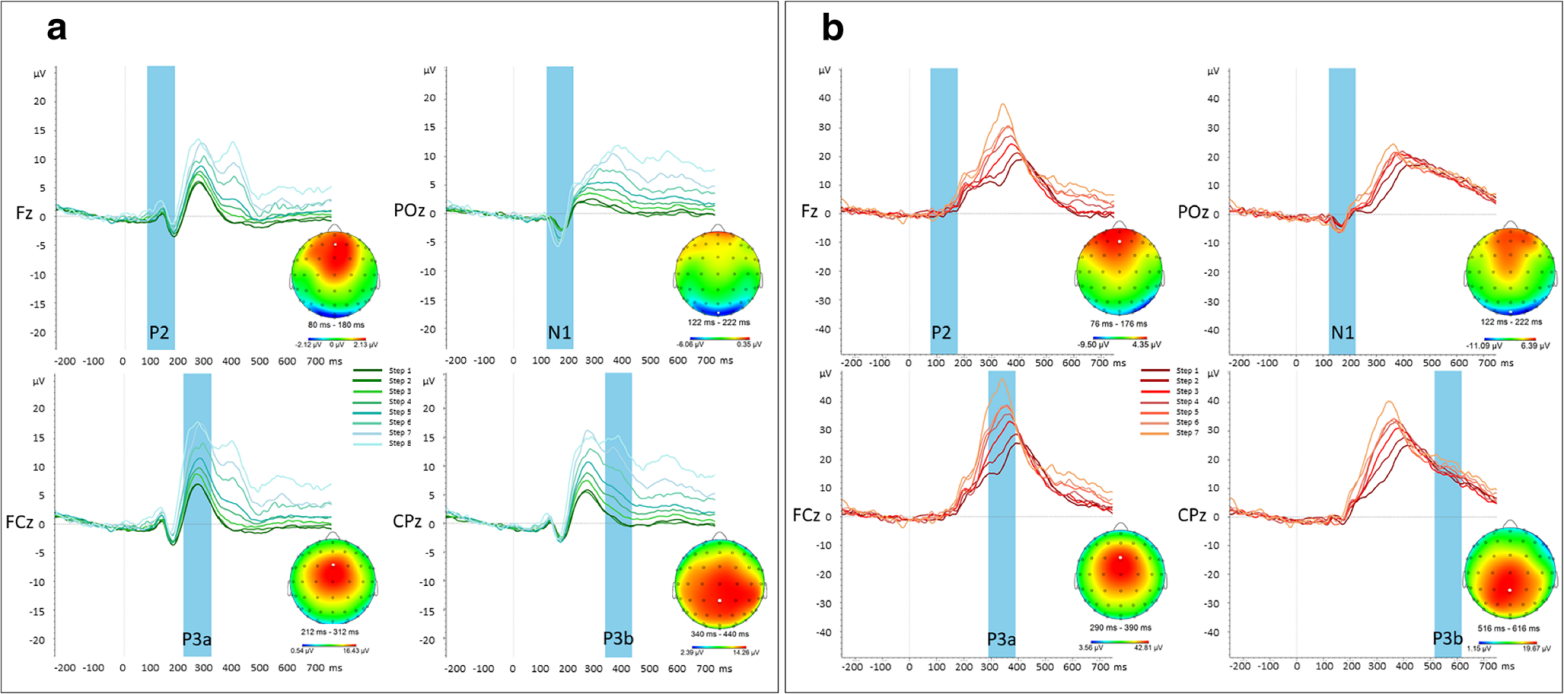
ERP components in response to successful feedback (**a**) and to burst feedback stimuli (**b**) per Inflation Steps 1–8. Topographic maps reflect spline maps showing a spherical 3D distribution of each component across the head (top view, nose is pointing upwards) in the interval of the blue transparent vertical bar. Zero ms indicates the time of stimulus onset. White dots on the topographical maps represent electrode locations at which each component was found to peak. (Color figure online)

To examine differences between the two conditions of interest in the amplitude and latency of event-related potentials (ERPs) in response to feedback stimuli in the EEG, we first examined effects of inflation step number on the ERPs by collapsing the data across (baseline, positive, and negative) forecasting conditions, following known practices for minimizing bias (Luck & Gaspelin, 2017). Visual inspection of changes in ERPs as a function of inflation step (separately for bubble bursts and successful inflations) in BrainVision Analyzer 2.1 (Brain Products GmbH, Munich, Germany) indicated an influence of inflation step on the amplitude and latency of four consecutive ERP components (see Fig. 2). Over frontal electrodes, and most clearly visible in the ERPs to successful feedback stimuli, the P2 was found to increase in amplitude and reduce in latency as a function of inflation step. Over occipital electrodes, the N1 was observed to increase in amplitude and was accompanied by a reduction in latency as a function of inflation step. Over centrofrontal electrodes, the P3a component was found to increase in amplitude and peak earlier with increasing inflation step. Finally, over centroparietal areas, and most clearly visible in the ERPs to successful feedback stimuli, the P3b component was found to increase in amplitude as a function of inflation step. Topographic maps and difference waves between inflation steps indicated that the P2 and P3b were also present in ERPs to bubble bursts, although the individual peaks of these components were not clearly visible in the grand average ERPs.

Against our expectations, in the current experiment no negative peak in the latency window of the FRN (~240 ms) and no signs of a component with a negative polarity signature in the spline maps and current source density (CSD) plots were identified to vary as a function of inflation step. Hence, the FRN was not included as a component in our analyses.^1^ Statistical testing of amplitude and latency differences of ERP components that were found to be sensitive to the increase of inflation step number follows in the Results section.

Peak latencies of the four components were determined separately per outcome condition (bursts, successful inflations) using visual inspection of the topography and amplitude of difference waves of ERPs to feedback stimuli presented at subsequent inflation steps. A window of 100 ms centered around the peak latency (−50 ms, +50 ms) of each ERP component was used for analysis. For ERPs time locked to positive feedback (successful inflations), these windows were P2 (80–180 ms); N1 (120–220 ms); P3a (210–310 ms); and P3b (340–440 ms). For ERPs in response to negative feedback stimuli (bubble bursts), these time windows were P2 (75–175 ms); N1 (120–220 ms); P3a (290–390 ms); and P3b (515–615 ms). For each component, the mean amplitude was calculated by taking the average amplitude of five electrodes—that is, the electrode at which the component peaked, and four surrounding electrodes on the basis of visual inspection of the topography, in order to increase signal to noise for statistical testing. The selected electrodes were in accordance with literature on the known distribution of these four components (N1, P2, P3a, P3b). For the P2, amplitude was calculated from Fz and the four surrounding electrodes (AFz, FCz, F1, and F2). For the N1, amplitude was averaged from POz and surrounding electrodes (Pz, Oz, PO3, and PO4). For the P3a, amplitude was averaged from FCz, and surrounding electrodes (Fz, Cz, FC1, and FC2). For the P3b, amplitude data were averaged from CPz and surrounding electrodes (Cz, Pz, CP1, and CP2). Subsequently, we extracted the averaged amplitude in microvolts (μV) and identified the peak latency of each component in the selected 100 ms intervals, separately per condition (baseline, positive, negative), stimulus (correct inflation, burst), and inflation step (1–8) for each participant. These peak latency and mean amplitude measures were used in the statistical analyses reported in the Results section.

Participants who were more risk averse produced less responses at higher inflation steps, resulting in fewer trials. A criterion of at least five trials for each ERP was used for inclusion in the study (Boudewyn, Luck, Farrens, & Kappenman, 2018). Supplementary Table S2 provides an overview of the number of participants and epochs that were included in the calculation of ERPs per condition and inflation step. The reduced signal to noise ratio at these higher inflation steps is taken into account by the mixed-model analysis (see below) that assigns statistical weight of condition cells as a function of the number of observations.

### Analysis procedure

All statistical analyses were conducted in R (R Core Team, 2011)*.* With respect to behavioural observations, we were first interested in differences in risk taking between blocks (i.e., the number of inflation responses following positive and negative economic forecasts). In line with the Petropoulos Petalas et al. (2017) study, we expected that participants would be inclined to take more risk following positive economic messages than following negative economic messages. We computed an analysis of variance (ANOVA) for a linear model using the *lm* function of the *stats* package (Version 3.5.2; R Core Team) to analyze differences in the number of inflation responses, as a function of block (baseline vs. positive information condition vs. negative information condition) and order (positive vs. negative information block presented first) as a well as their two-way interaction. Post hoc contrasts using the Tukey method for multiple comparisons were used to test pair-wise comparisons between blocks.

In addition, and similar to the Petropoulos Petalas et al. (2017) study, we expected the effect of positive and negative forecasting on risk taking to be consistent over the course of their respective blocks. We therefore tested whether the rate of change in risk taking scores across trial number for each condition was significant. For these simple linear models, *p* values for the main effects of block and trial number were estimated using conditional *F* tests (Type III sum of squares), as applied in the *Anova* function (package *car*, Version 2.0-21; Fox, Friendly, & Weisberg, 2013).

In view of the nested nature of the RT and EEG data (as we obtained observations from repeated measures both within participants and trials), we chose a (generalized) linear mixed-effects model for the analyses. This type of model allows viewing the data without necessarily aggregating at the trial or at the participant levels, thus resulting in lower unexplained variance and higher statistical power to account for effects at both levels (condition, step) of the analysis. In addition, mixed models handle missing data more appropriately compared with traditional methods, such as the repeated-measures ANOVA (Baayen, Davidson, & Bates, 2008; Barr, Levy, Scheepers, & Tily, 2013; Vaughn, 2008). We therefore used the *(g)lmer* function of the *lme4* package (Version 1.1-7; Bates, Mächler, Bolker, & Walker, 2015) in R to investigate differences in RT and EEG data between the two main experimental conditions per number of inflation step. Following Barr (2013), a maximal random-effects model structure was used where possible, including a per-participant random adjustment to the fixed intercept and a per-participant random adjustment to the slopes of predictors varying within subjects—in this case, number of inflation step and condition. All possible random terms (i.e., random intercept and slope) across random effects were included, unless otherwise specified. For these generalized linear mixed-effects models, *p* values were determined using bootstrapped likelihood ratio tests, as implemented in the *anova* function (package *stats4*, Version 3.0.1; R Core Team, 2011). All confidence intervals reported are at 95%, unless otherwise specified.

We examined RT data to investigate effects of inflation step and economic forecast condition. Following the advice by Baayen and Milin (2010), we first removed responses below 100 ms, as they most likely reflect accidental button presses or participants holding down the button to speed up the trial. Overall, 5.1% of all observations (1,453 out of 28,439 in total) were identified as unjustifiably fast (<100 ms), and they were removed from the data set. Albeit the data were skewed, it was decided to not transform the data, to ease results interpretation. Modern multilevel approaches in analyses of reaction times suggest that distributional skewness is not as much of a problem compared with the problem of interpreting logarithmically transformed data (Lo & Andrews, 2015). A multilevel mixed-model analysis was used in R, as implemented using the *lmer* function (LME4 package). We first tested whether a random effects structure was warranted and compared a *lm()* to a *lmer()* model structure. As expected, adding the random structure improved the model fit. Following the advice by Bates et al. (2015), we then ran a maximum model structure that included main effects for the factors of step (i.e., inflation step number) and condition (i.e., valence of forecast) as well as their interaction—and random slopes and intercepts for the Step × Condition interaction effect, as well as for the (random) effects of trial number and participant number. This maximal structure model did not converge; therefore, in accordance with Barr (2013), steps were then taken to reduce the processing power (i.e., first, by trying different data optimizers) and the model’s complexity (i.e., subsequently, by gradually reducing the model’s structure until a model fit the data best). The best fitting model (REML criterion at convergence: 215,706) excluded number of inflation step from the random structure, and included a random effects structure only for the main effect of condition, with correlated intercept and slopes. By consecutively adding parameters to the model, we tested whether they significantly improved the model fit. Adding step as a mixed factor significantly improved the model fit (*p* < .001, BIC fit/full = 216385/216121), and so did condition (*p* < .001, BIC fit/full = 216142/216121), as well as the interaction term (*p* < .001, BIC fit/full = 216212/216121).

Concerning the EEG data, we were interested in the amplitudes and latencies of the four components that were found to vary as a function of the increase in inflation step in the BART (see Fig. 2). Similarly to analyzing RTs, due to the small number of responses to inflate beyond inflation Step 8, we only looked at differences until and including Inflation Step 8. Moreover, and because the first inflation never resulted in a bubble burst, all analyses for bubble burst were limited to Inflation Steps 2–8. We ran linear mixed-model analyses to test for effects of inflation step and forecasting condition on ERP amplitude and latency, for each of the four components of interest (P2, N1, P3a, and P3b) and for each feedback event type (successful inflations and bubble bursts). Each model included fixed effects for step and condition, as a well as their two-way interaction. As random effects, we included the intercepts per participants, and for the main effects of step and condition, we allowed per-participant random slopes. This maximal random-effects model structure was used in all cases, and visual inspection of residual plots did not reveal homoscedasticity or deviations from normality. However, for modeling differences in latency of the P3b in the case of bubble bursts, we had to eliminate the random correlation and random intercept terms, as a maximal structure failed to converge. The best fitting model included fixed effects for inflation step number and condition, as well as the interaction term and per-participant random slopes. As mentioned earlier, *p* values were obtained by likelihood ratio tests of the full model with the effect in question, compared against the model without the effect in question.

## Results

### Behavioural responses

#### Risk taking (bubble inflations)

Risk-taking scores were calculated as the maximal number of bubble inflations performed by the participant for each trial, until the participant decided to collect or a bubble explosion had occurred. All data were normally distributed, and no outliers had to be removed. Participants inflated the bubble on average 6.72 times in the baseline (*SD* = 1.74, *SE* = .059), 6.68 times in the negative forecasting condition (*SD* = 1.91, *SE* = .064), and 7.71 times in the positive forecasting condition (*SD* = 1.81, *SE* = .066).

**Fig. 3:**
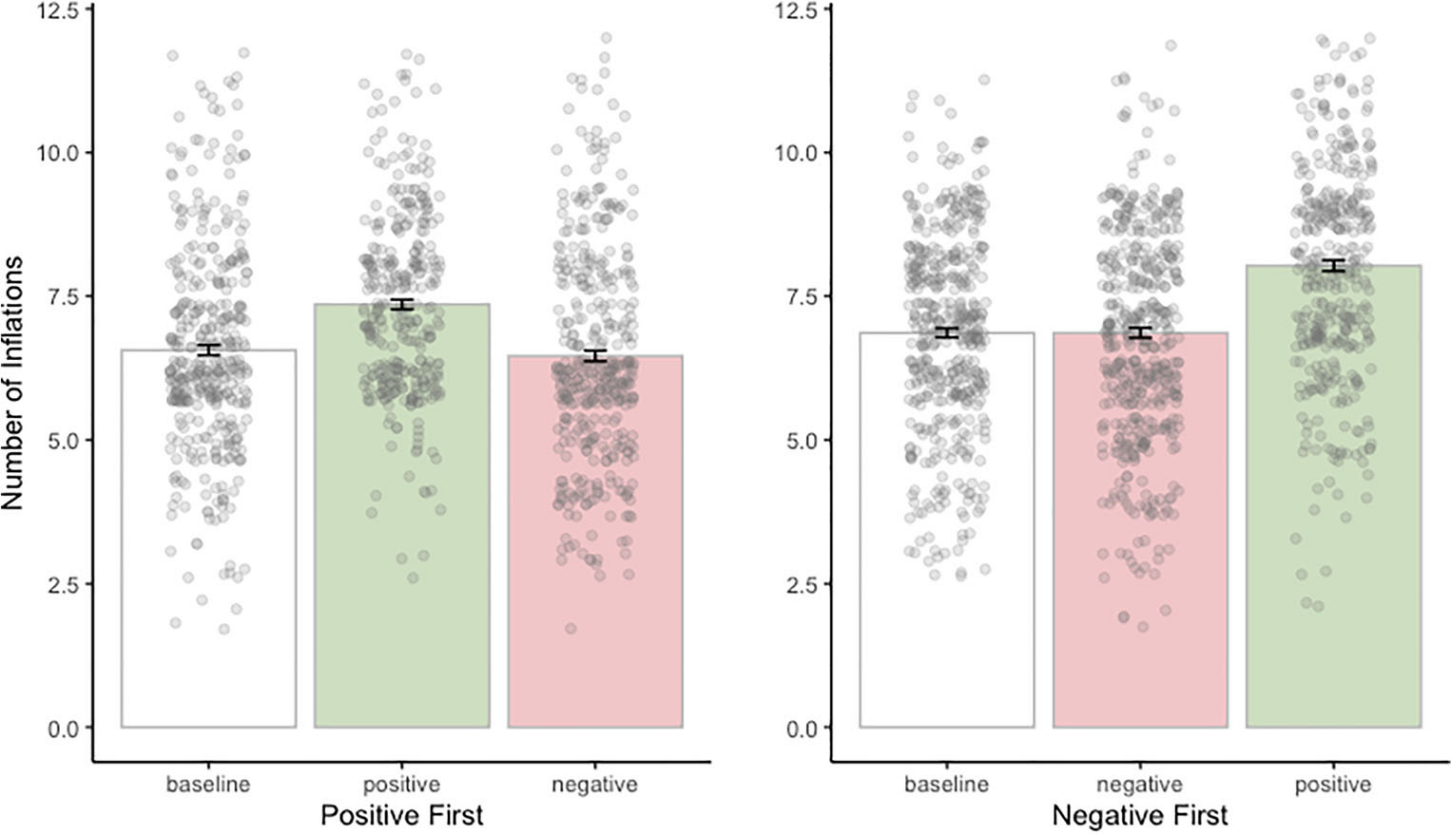
Number of inflation responses (risk taking) in the baseline (white) and for each of the two forecasting conditions (red for negative and green for positive forecasting), plotted separately for the difference in order of presenting the positive and the negative message forecasts. Whiskers indicate standard error. (Color figure online)

We used an ANOVA to analyze differences in the mean number of inflation responses per experimental condition, with experimental block (baseline vs. positive information condition vs. negative information condition) and order (positive vs. negative information condition presented first) as a well as their two-way interaction as predictors, and with number of inflations as the dependent variable. Results of the linear mixed-effects model to investigate differences in risk taking as a consequence of economic forecasting indicated a main effect of block, *F*(2, 2498) = 81.59, *p* < .001, partial η^2^ = .015, and a main effect of order on the number of inflation responses, *F*(1, 2498) = 38.07, *p* < .001, partial η^2^ = .001. The two-way Block × Order interaction was not significant (*p* = .108). These findings suggest that although the two counterbalancing groups performed differently on the BART, the order in which conditions were administered did not influence the main effect of block (i.e., the effect of the economic forecast; see Fig. 3)*.* Post hoc contrasts using the Tukey method for multiple comparison showed a significant estimate difference of Δ*M =* 1.02 inflations, *SE* = .09, *t*(2,498) = 11.41, *p* < .001, partial η^2^ = .061, 95% CI [−1.03 , −.81], meaning that participants took more risk in the positive and less risk in the negative forecasting conditions, and this difference was statistically significant. The difference in inflation scores between baseline and positive forecasting condition was also significant, Δ*M =* .97 inflations, *SE* = .09, *t*(2,498) = 10.73, *p* < .001, 95% CI [−1.19, −.76]). Last, the difference in inflation scores between baseline and negative forecasting condition was insignificant (*p* = .832). See Fig. 3 for a schematic illustration of these results.

The number of “bubbles collected” (instances in which participants opted for collecting gains and thus to stop inflating the bubble) was greater than the number of “bubble bursts” (instances in which the bubble exploded) at the baseline (*M*_Collected_ = 42.8, *SD*_Collected_ = 0.29 and *M*_bursts_ = 37.2, *SD*_*bursts*_ = 0.27) and at the negative forecasting condition (*M*_Collected_ = 45, *SD*_Collected_ = 0.30 and *M*_bursts_ = 35, *SD*_bursts_ = 0.26); however, this was not the case in the positive forecasting condition (*M*_Collected_ = 37.5, *SD*_Collected_ = 0.26 and *M*_bursts_ = 42.4, *SD*_bursts_ = 0.28). The difference in bubble collected across the three blocks was significant, *F*(1, 28436) = 21.34, *p* < .001, partial η^2^ = .007, while the difference in bubble bursts was not.

**Fig. 4:**
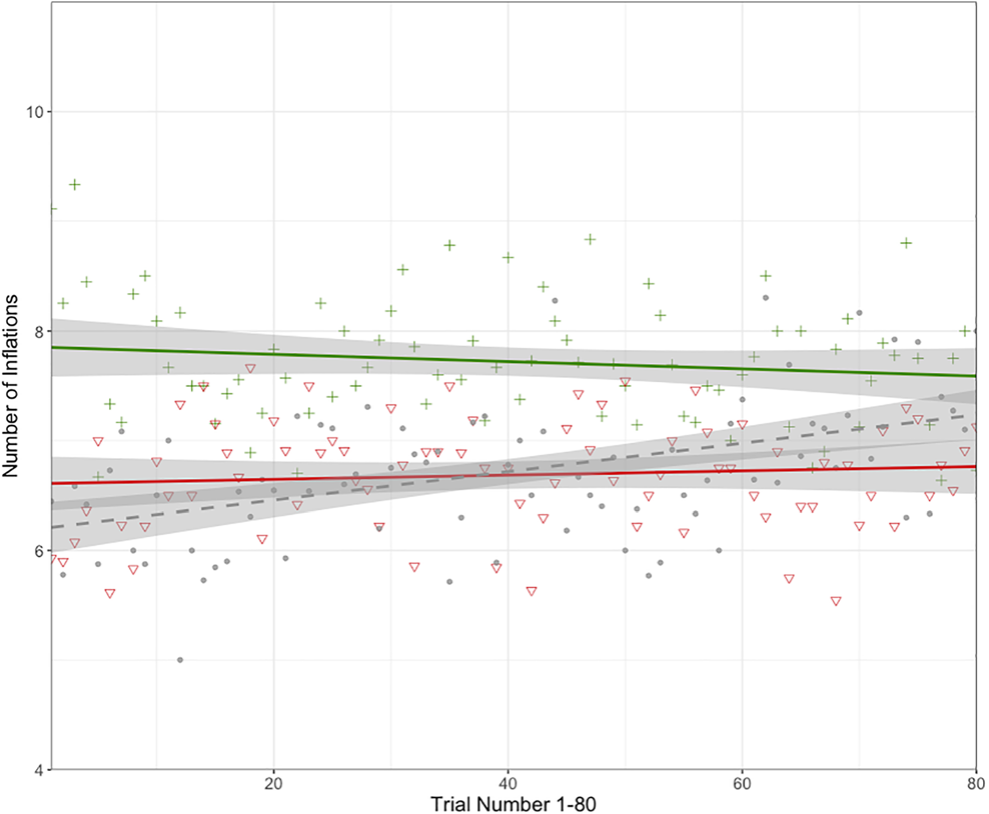
Risk taking in the BART as a function of Trials 1–80, for the baseline block (dashed line) and each of the experimental blocks following positive (top solid line) and negative (bottom solid line) economic forecasting. Individual points (baseline = small solid dots; negative forecasting = triangle symbol; positive forecasting = cross symbol) signify aggregate responses across trials. Shaded regions represent 95% confidence intervals

To investigate whether the effect of the message forecast on risk taking is resilient over the course of trials in the BART, we looked at the rate of change in risk taking across all 80 trials per experimental condition. Although participants’ average risk taking increased significantly as a function of trial numbers 1–80 in the baseline (*R*^2^ = .029, *b* = .013, *p* < .001), there were no significant changes in risk taking throughout the 80 trials of the experimental blocks, neither in the negative (*R*^2^ < .001, *b* = .001, *p* = .477) nor in the positive (*R*^2^ = .001, *b* = −.003, *p* = .256) forecasting condition (see Fig. 4). The absence of a significant change in risk taking in the positive and negative experimental blocks denotes that the effect of the positive and negative forecast manipulation was stable over the course of these respective block (see Fig. 4 for a graphical representation of risk taking over trials). The increase in risk taking in the baseline may reflect a learning effect, in the sense that participants may be more anxious or careful in the beginning of the baseline block and gain confidence over trials, manifested as an increase in risk taking.

#### Reaction times (RTs)

We expected slower responses for successive inflations steps, reflecting higher uncertainty for increasingly riskier decisions (Petropoulos Petalas et al., 2017; Pleskac & Wershbale, 2014). Furthermore, we expected the rate of change in RTs to be steeper for the negative versus the positive forecasting condition, reflecting the increasingly stronger uncertainty in case of a negative economic forecast. Table 1 presents a summary of descriptive statistics of RTs per condition and inflation step.

**Table 1:** Descriptive statistics of RTs (in milliseconds) per forecasting condition and at each inflation step number 1-11.

Inflation step number	Negative forecasting			Positive forecasting		
	*n*	Mean RT (*SD*)	*SE*	*n*	Mean RT (*SD*)	*SE*
1	1594	472 (405)	10.17	1597	488 (432)	10.82
2	1418	446 (369)	9.8	1450	474 (458)	12.02
3	1224	465 (422)	12.05	1297	482 (465)	12.91
4	980	426 (404)	12.92	1133	464 (409)	12.14
5	654	443 (514)	20.09	878	421 (402)	13.58
6	444	455 (559)	26.52	634	448 (419)	16.77
7	245	509 (803)	51.31	390	414 (418)	21.19
8	85	422 (346)	37.51	184	487 (630)	46.46
9	52	1045 (1899)	263.33	91	549 (700)	73.4
10	19	413 (258)	59.15	38	1109 (1682)	272.82
11	8	907 (1503)	531.55	7	3189 (3858)	1458.21

*Note*. Descriptive statistics on aggregate data across all steps, trials, and participants. RTs smaller than 100 ms have been removed (see text)

**Fig. 5:**
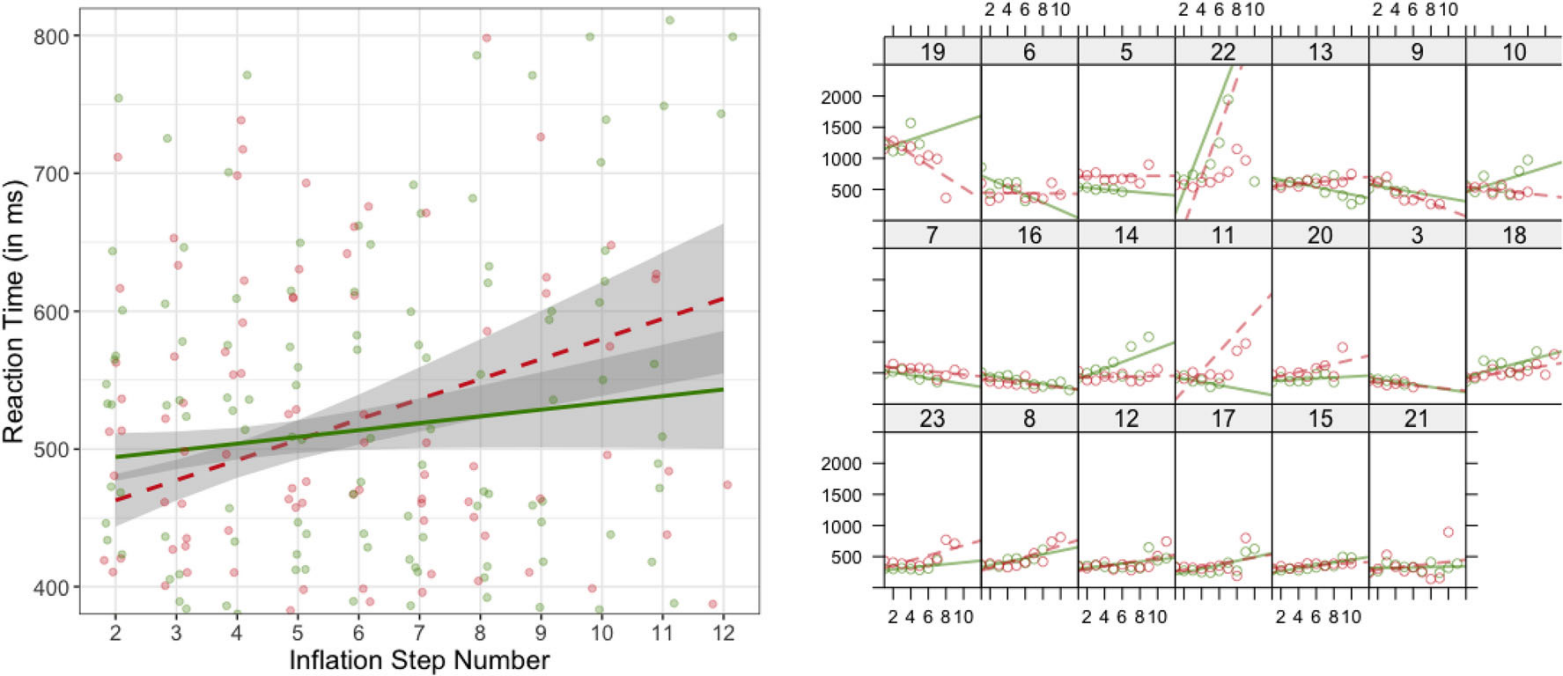
Linear trend of the reaction times (RTs) in milliseconds per inflation step number, for the positive (solid green line) and for the negative (dashed red line) forecasting conditions, both at the aggregate (left side) and at the individual level (right side). Shadowing represents 95% CIs. (Color figure online)

Likelihood ratio tests indicated a significant effect of step, *t*(21.67) = 14.45, *p* < .001, *SE* = 48.07, meaning that RTs were significantly slower as the number of inflation steps became higher. The effect of the condition was also significant, *t*(113.36) = 8.36, *p* < .001, *SE* = 15.94, thereby confirming that negative forecasting elicits slower responses in the decision to inflate, compared with positive forecasting. Last, there was a significant interaction effect, *t*(14,338.12) = 20.59 *p* < .001, *SE* = 14.55, meaning that the rate of change in RTs differed significantly between the two experimental conditions. In all, the analysis of reaction times showed slower RTs for successively riskier decisions (i.e., an effect of step) and further suggested this effect was stronger for the case of negative economic forecasting (see Fig. 5 for a schematic presentation of these differences).

### EEG results

#### ERP amplitudes

**Fig. 6:**
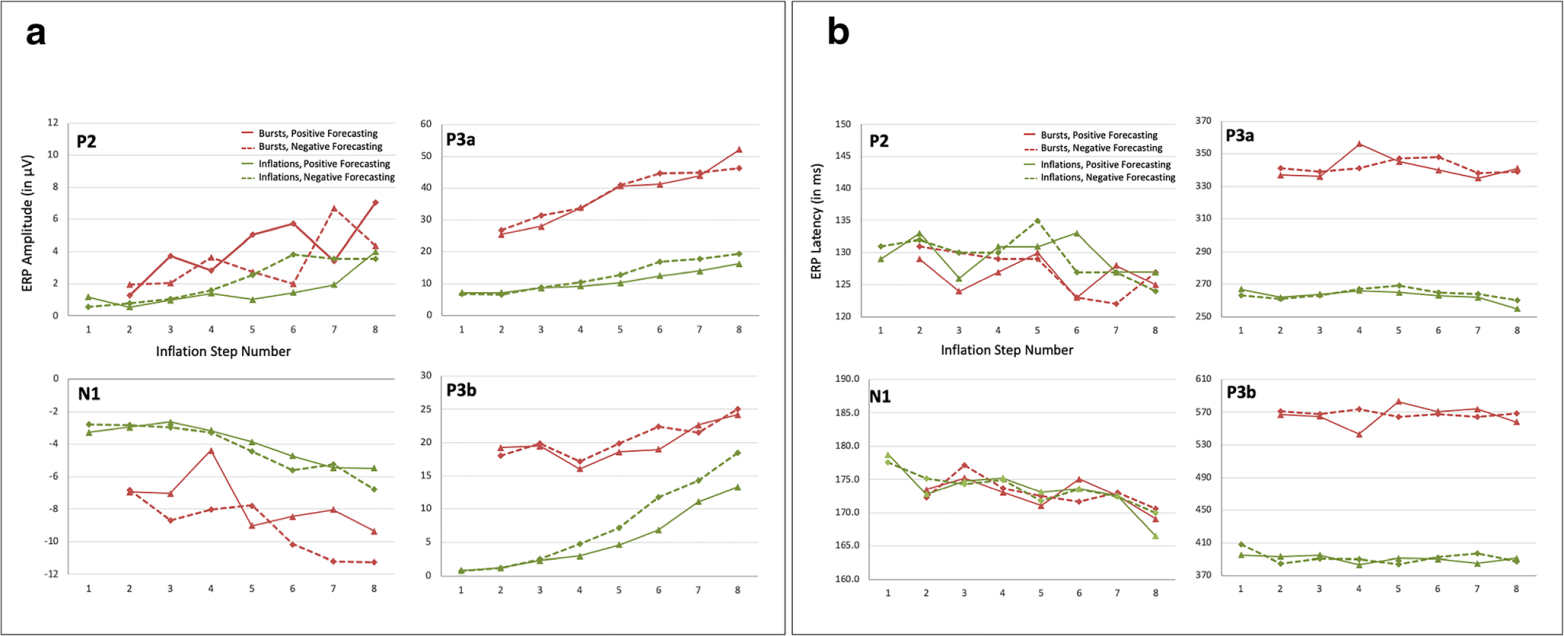
Summary of ERP amplitude (**a**) and latency **b**) differences as a function of Inflation Steps 1–8. Green (red) lines correspond to feedback from successful inflations (bubble bursts); solid (dotted) lines correspond to positive (negative) economic forecasting conditions, accordingly. (Color figure online)

We first looked at differences in ERP amplitudes of the P2, N1, P3a, and P3b components to feedback screens, indicating successful bubble inflations and bubble bursts. Amplitudes of most of these components differed significantly as a function of the increase in inflation step number and also as a function of the forecasting condition. For an overview of the amplitude of the four components in positive and negative forecasting blocks, see Table 2 and Fig. 6. Also see Figs. S1, S3, and S4 in the Supplementary Materials. Figure S1 presents an overview of the component amplitudes with detailed information on the data points of individual participants. Figures S3 and S4, respectively, present the ERPs to successful inflations and bubble bursts separately for positive and negative forecasting conditions. Below, we detail the outcomes of the analyses per component and feedback screens.

- ***P2***. As can be seen in Figs. 6 and S3, for successful inflations, the amplitude of the P2 component to positive feedback stimuli generally increased as a function of inflation step, χ^2^(7) = 28.83 , *p* < .001, a contrast of Δ*Μ* = 3.01 μV (*SE* = 3.42) between Step 1 and Step 8. The main effect of condition, χ^2^(1) = 6.79, *p* = .009, and the Inflation Step × Condition interaction, χ^2^(7) = 17.57, *p* = .014, were also significant. At higher inflation steps (in particular, Steps 5, 6, and 7), negative forecasting resulted in higher P2 amplitudes compared with positive forecasting, by Δ*Μ* = 0.54 μV (*SE* = 0.21). For bubble bursts, a trend towards an effect of step, χ^2^(6) = 11.51, *p* = .073, was found, reflecting an increase in P2 amplitude by Δ*Μ* = 3.12 μV (*SE* = 1.52) across Inflation Steps 2–8. The main effect of condition was significant, χ^2^(1) = 5.04, *p* = .024, indicating that on average the positive forecasting message resulted in higher P2 amplitude by Δ*Μ* = 1.40 μV (*SE* = 0.66). The Inflation Step × Condition interaction was also significant, χ^2^(6) = 20.01, *p* < .01, indicating that the stronger P2 amplitude for the positive forecasting condition was found for most steps (3, 5, 6, and 8), but not for other steps (2, 4, and 7).
- ***N1***. For successful inflations, the amplitude of the N1 to positive feedback stimuli increased significantly at higher inflation steps, χ^2^(7) = 34.33, *p* < .001, a contrast of Δ*Μ* = 2. 91 μV (*SE* = 3.64) between Step 1 and Step 8. The main effect of condition and the Step × Condition interaction effect were not significant. For bubble bursts, a similar increase in N1 amplitude was found as a function of step number, χ^2^(6) = 21.55, *p* < .01; the difference in magnitude of the N1 component between Steps 2–8 was Δ*Μ* = −2.73 μV (*SE* = 1.74). In addition, we found a trend towards a main effect of condition, χ^2^(1) = 3.79 , *p* = .051, showing greater amplitude for the negative (*M* = −8.93 μV, *SE* = 1.05, 95% CI [−11.10 , −6.77]) compared with the positive (*M* = −7.76 μV, *SE* = 0.82, 95% CI [−9.45 , 6.07]) forecasting condition; a contrast of Δ*Μ* = −1.16 μV (*SE* = 0.62). The interaction effect of Step × Condition was also significant, χ^2^(6) = 19.54, *p* < .01, indicating that the stronger amplitude of the N1 in the negative forecasting condition varied (unsystematically) over inflation steps.

**Table 2:** Feedback-locked ERP amplitudes across Inflation Steps 1–8 and per forecasting condition

Inflation step number	Successful inflations								Bubble bursts							
	ERPs in response to negative forecasting				ERPs in response to positive forecasting				ERPs in response to negative forecasting				ERPs in response to positive forecasting			
	P2	N1	P3a	P3b	P2	N1	P3a	P3b	P2	N1	P3a	P3b	P2	N1	P3a	P3b
1	0.55 (1.67)	−2.78 (2.28)	6.85 (3.91)	0.68 (2.15)	1.19 (1.40)	−3.26 (2.38)	7.14 (3.24)	0.79 (2.36)	–	–	–	–	–	–	–	–
2	0.80 (2.08)	−2.80 (1.80)	6.66 (3.83)	1.19 (1.18)	0.55 (1.57)	−2.95 (1.62)	7.22 (3.49)	1.18 (2.46)	1.13 (5.11)	−6.81 (4.60)	26.64 (16.58)	18.04 (7.02)	1.96 (3.34)	−6.94 (5.55)	25.43 (14.47)	19.25 (7.00)
3	1.06 (1.83)	−2.94 (2.58)	8.73 (3.30)	2.43 (2.33)	0.97 (1.83)	−2.62 (2.05)	8.70 (3.98)	2.26 (2.50)	3.74 (4.43)	−8.70 (5.25)	31.24 (15.63)	19.93 (8.96)	2.08 (4.57)	−7.03 (4.58)	27.92 (15.96)	19.50 (9.48)
4	1.62 (2.78)	−3.27 (2.36)	10.42 (4.26)	4.77 (4.17)	1.41 (2.43)	−3.14 (1.98)	9.21 (4.28)	2.94 (2.51)	2.82 (6.05)	−8.04 (4.66)	33.65 (12.88)	17.20 (6.82)	3.63 (5.47)	−4.37 (4.30)	33.75 (14.28)	16.01 (9.46)
5	2.56 (2.46)	−4.44 (3.35)	12.73 (5.80)	7.17 (5.16)	1.02 (2.69)	−3.85 (2.66)	10.33 (5.58)	4.61 (3.89)	5.06 (7.40)	−7.98 (5.27)	40.88 (19.04)	19.93 (6.71)	2.73 (7.04)	−9.04 (3.97)	40.71 (20.01)	18.60 (7.78)
6	3.84 (5.29)	−5.61 (4.42)	16.85 (8.73)	11.74 (10.03)	1.46 (2.69)	−4.72 (2.83)	12.47 (5.96)	6.87 (4.54)	5.73 (5.74)	−10.16 (4.72)	44.59 (16.91)	22.44 (7.88)	2.01 (4.53)	−8.47 (3.79)	41.23 (16.47)	19.01 (8.29)
7	3.56 (3.54)	−5.25 (3.72)	17.74 (8.78)	14.32 (8.36)	1.98 (2.70)	−5.45 (3.12)	13.89 (6.29)	11.12 (5.04)	3.41 (3.77)	−11.22 (7.20)	44.74 (11.82)	21.55 (6.43)	6.70 (6.04)	−8.04 (6.78)	43.92 (17.84)	22.69 (10.28)
8	3.54 (2.37)	−6.78 (3.22)	19.29 (5.77)	18.46 (7.18)	3.99 (3.52)	−5.47 (4.24)	16.18 (8.26)	13.36 (7.16)	7.07 (4.72)	−11.28 (547)	46.21 (13.26)	25.07 (7.50)	4.37 (9.41)	−9.37 (6.65)	52.12 (20.55)	24.22 (10.08)

*Note*. Mean amplitude (in μ*V*) and standard deviation (*SD*) as directly extracted from BrainVision Analyzer. The first inflation never led to a bubble burst (see text)

- ***P3a***. For successful inflations, the amplitude of the P3a component was found to increase as a function of inflation step, χ^2^(7) = 53.51, *p* < .001; a contrast of Δ*Μ =* 1.58 μV (*SE* = 0.22) from Steps 1–8. The main effect of condition was not significant, but the Step × Condition interaction effect was, χ^2^(7) = 39.07, *p* < .001, indicating a stronger amplitude of the P3a in the negative forecasting condition at increasing inflation steps (5–8). For bubble bursts, the main effect of step was significant, χ^2^(6) = 91.65, *p* < .001; the amplitude of the P3a component increased across Inflation Steps 2–8 by 25.40 μV (*SE* = 3.43). Both the main effect of condition and the interaction effect of Step × Condition were insignificant.
- ***P3b***. For successful inflations the amplitude of the P3b component increased as a function of inflation step, χ^2^(7) = 100.23 , *p* < .001; a contrast of Δ*Μ =* 15.08 μV (*SE* = 1.62) between Steps 1 and 8. The main effect of condition was not significant, but the interaction effect of Step × Condition was significant, χ^2^(7) = 37.90 , *p* < .001, indicating a stronger amplitude of the P3b in the negative forecasting condition at increasing inflation steps (4–8). For bubble bursts, a significant main effect of inflation step, χ^2^(6) = 15.66, *p* = .015, was found, showing that the amplitude of the P3b component across Inflation Steps 2–8 increased by 7.12 μV (*SE* = 2.21). Both the main effect of condition and the interaction effect were insignificant.

#### ERP latencies

We also looked at variations in peak latency of the P2, N1, P3a, and P3b components, again separately for successful inflations and for bubble burst feedback screens. Latencies of most of these components differed significantly as a function of the increase in inflation step number, but not as a function of the economic forecasting condition, albeit interaction effects were noticed. See Table 3 as well as Fig. 6 for an overview of these differences. Also see Fig. S2 in the Supplementary Materials, which presents an overview of the component peak latencies with detailed information on the data points of individual participants.

- ***P2***. For successful inflations, we found a marginally significant effect in the latency of the P2 over inflation steps, χ^2^(7) = 14.039, *p* = .050, while the main effect of condition was insignificant (*p* = .726). However, there was a trend towards a Step × Condition interaction, χ^2^(7) = 12.901, *p* = .074, reflecting a different pace of variation in P2 latency between the two experimental conditions of interest. For bubble bursts, the increase in Step Numbers 2–8 did not significantly affect the latency of the P2 component (*p* = .165). Furthermore, neither the main effect of condition (*p* = .580) nor the interaction effect of Condition × Step (*p* = .272) were found to be significant.
- ***N1***. For successful inflations, the increase in Step Numbers 1–8 significantly reduced the latency of the N1 component, χ^2^(7) = 23.37, *p* < .001; a mean difference of −9.26 ms (*SE* = 2.43) across Inflation Steps 1–8. The main effect of condition (*p* = .527) and the interaction effect of Inflation Step × Condition (*p* = .209) were not significant. For bubble bursts, the latency of the N1 peak varied over inflation steps, χ^2^(6) = 15.953, *p* = .014, revealing a similar pattern as the N1 latency to feedback of successful inflation; the mean difference in the latency of N1 between Inflations Steps 2–8 was −3.47 ms (*SE* = 1.91). Neither the main effect of condition (*p* = .822) nor the Inflation Step × Condition interaction (*p* = .252) was significant.
- ***P3a***. For successful inflations, neither the main effects of condition (*p* = .777) and step (*p* = .454) nor the interaction effect between Step × Condition (*p* = .625) were significant. For bubble bursts, neither the main effects of step (*p* = .455) and condition (*p* = .636) nor the interaction between Step × Condition (*p* = .593) were significant.
- ***P3b*.** For successful bubble inflations, the latency of the P3b was found to vary over inflation steps, χ^2^(7) = 14.49, (*p* = .043). The main effect of condition (*p* = .387) was insignificant; however, the interaction effect between Step × Condition was significant, χ^2^(7) = 14.41, *p* = .044, reflecting different fluctuations in P3b latency between conditions. For bubble bursts, no significant main effect of step (*p* = .922) or interaction effect with condition (*p* = .673) were found.

**Table 3:** Feedback-locked ERP latencies across Inflation Steps 1–8 and per forecasting condition

	Successful inflations								Bubble bursts							
Inflation step number	ERPs in response to negative forecasting				ERPs in response to positive forecasting				ERPs in response to negative forecasting				ERPs in response to positive forecasting			
	P2	N1	P3a	P3b	P2	N1	P3a	P3b	P2	N1	P3a	P3b	P2	N1	P3a	P3b
1	131 (10.80)	178 (8.59)	263 (12.97)	408 (23.02)	129 (12.65)	179 (8.35)	267 (14.39)	395 (20.79)	–	–	–	–	–	–	–	–
2	132 (8.87)	175 (7.45)	261 (16.45)	385 (16.36)	133 (9.39)	173 (8.80)	262 (16.82)	393 (22.68)	131 (10.22)	172 (7.67)	341 (45.51)	571 (55.29)	129 (11.40)	173 (6.31)	337 (43.66)	567 (54.45)
3	130 (10.60)	174 (8.01)	263 (13.70)	391 (20.61)	126 (9.87)	175 (8.09)	264 (11.71)	395 (23.51)	130 (10.64)	177 (7.08)	339 (37.08)	568 (38.15)	124 (12.59)	175 (5.88)	336 (46.78)	565 (49.13)
4	130 (11.47)	175 (6.96)	267 (15.41)	390 (17.06)	131 (9.17)	175 (8.41)	266 (18.04)	383 (17.96)	129 (14.40)	174 (7.21)	341 (33.85)	574 (31.70)	127 (12.28)	173 (6.74)	356 (31.14)	543 (38.36)
5	135 (8.00)	172 (7.66)	269 (18.67)	384 (21.80)	131 (10.91)	173 (6.13)	265 (16.06)	391 (23.77)	129 (9.74)	172 (4.83)	347 (30.44)	564 (45.32)	130 (11.40)	171 (5.76)	345 (35.61)	583 (59.14)
6	127 (9.66)	174 (5.66)	265 (18.89)	392 (23.22)	133 (10.37)	174 (7.09)	263 (17.05)	390 (20.40)	123 (14.24)	172 (7.49)	348 (27.19)	568 (45.85)	123 (14.23)	175 (7.77)	340 (29.03)	571 (45.59)
7	127 (13.94)	172 (5.75)	264 (20.12)	397 (24.90)	127 (12.94)	172 (6.56)	262 (13.13)	385 (18.91)	122 (13.57)	173 (5.88)	338 (26.73)	564 (38.11)	128 (8.79)	172 (6.51)	335 (33.84)	574 (51.63)
8	124 (24.33)	169 (6.39)	260 (19.96)	387 (18.91)	127 (12.21)	166 (6.29)	255 (20.91)	391 (23.22)	127 (10.31)	170 (7.28)	339 (21.67)	569 (42.22)	125 (11.28)	169 (6.25)	341 (24.10)	558 (46.68)

*Note.* Mean latency (in ms) and standard deviation (*SD*) as directly extracted from BrainVision Analyzer. The first inflation never led to a bubble burst (see text)

## Discussion

The goal of the present study was to investigate the neurophysiological basis of economic decision-making in the BART and to find converging evidence from ERPs for the idea that economic forecasts can influence people’s internal model of the economic reality and their subsequent financial decision-making. We hypothesized that beliefs regarding the BART economy would be reflected in electrophysiological brain responses that are sensitive to risk taking in the BART. In accordance with this aim, we found four ERP components (P2, N1, P3a, and P3b) to be sensitive to the level of risk taking in the BART. Furthermore, and most importantly, messages about possible changes in the BART economy were found to systematically influence the amplitude and/or the latency of these components, supporting the theoretical idea that economic forecasting may change people’s mental model of the economy and influence their perception of risk in financial decision-making. Thereby, the findings of the current study corroborate the self-fulfilling prophecy effect in economic forecasting, which states that economic forecasts may influence people’s beliefs about the economy and their consecutive financial decision-making, thus effectuating the economic prophecy that was forecasted in the first place.

The behavioural outcomes of the present study largely replicate the results of the Petropoulos Petalas et al. (2017) study. Similar to that study, we found that economic messages forecasting a potentially negative development in the economy resulted in participants taking less risk in the BART than following messages forecasting a possible positive economic development. The changes in financial risk taking were found to be substantial: more than one inflation on average in the positive news condition on an average of 7.2 inflations per trial. Furthermore, in statistical terms, the effect size of the difference in number of inflations per block can be considered a moderate effect (Abelson, 1985; Ferguson, 2009). In addition, analysis of the forecasting effect across trials indicated that that the effect set in immediately following the economic messages and remained stable throughout the block (see Fig. 4). As previously argued (Petropoulos Petalas et al., 2017), these latter two findings likely follow from the probabilistic risk function of the BART that was unknown to the participants (as is the case for the real economy), which makes it difficult for participants to determine if and when an economic forecast has become a reality, and to correct potentially false beliefs through experience sampling (H. Zhang, Paily, & Maloney, 2015). Last, the analysis of reaction times showed prolonged RTs for successively riskier decisions (i.e., an effect of step) and further suggested this effect was stronger for the case of negative economic forecasting. In line with Petropoulos Petalas et al. (2017), this finding suggests that participants’ uncertainty increased with inflation step and was enhanced by negative economic forecasts relative to positive forecasts.

The electrophysiological effects of economic forecasting in the present study play a crucial role in the sense that they corroborate the interpretation that economic forecasts led to a change in the participants’ mental model of the (BART) economy, which in turn influenced their financial decision-making. Overall, we found that the amplitudes of P2, N1, P3a, and P3b to feedback stimuli increased in amplitude with increasingly riskier gambles and that negative economic forecasts further accelerated this increase in component amplitude, relative to positive economic forecasts. The effect of economic forecasting was not the same for each component, however, and was found to vary with the type of feedback stimulus (success or burst) that was presented.

### P2

At frontocentral electrode sites feedback stimuli indicating a successful inflation evoked a P2 with a peak around 130 ms. A P2 with a similar frontocentral topography but without a clear peak (as the ERP transitioned in a P3a; see, e.g., San Martín, Appelbaum, Pearson, Huettel, & Woldorff, 2013) was observed following feedback stimuli, indicating a burst. The P2 has been associated with the evaluation of stimulus relevance (Chen, Zhang, Zhong, Hu, & Li, 2013; Potts, 2004) and the recruitment of attentional resources (Carretié, Hinojosa, Martín-Loeches, Mercado, & Tapia, 2004; Carretié et al., 2001a, b; Chen et al., 2013) to potentially threatening and emotionally relevant stimuli such as faces with negative (e.g., fearful or angry) emotional expressions (Chen et al., 2012; Eimer & Holmes, 2007; Moser, Huppert, Duval, & Simons, 2008; Wang, Liu, & Yan, 2014; Yang, Yuan, & Li, 2012), images evoking strong negative emotions such as fear or disgust (Carretié et al., 2001a, b; Chen et al., 2013; Delplanque, Lavoie, Hot, Silvert, & Sequeira, 2004; Yuan et al., 2007), and cues signaling an impeding threat (Gibbons, Schnuerch, & Stahl, 2016; Rossignol, Philippot, Douilliez, Crommelinck, & Campanella, 2005; Bublatzky & Schupp, 2012). Furthermore, studies examining feedback processing in gambling tasks (Schuermann, Endrass, & Kathmann, 2012; West et al., 2014) have found P2 responses to feedback stimuli to increase in amplitude with more negative outcomes (e.g., S. Xu et al., 2011) and to grow in size with increasing risk (i.e., with increasing outcome variance (Kiat, Straley, & Cheadle, 2016; Goyer, Woldorff, & Huettel, 2008). In line with these findings, the amplitude of the P2 to feedback stimuli in the current study was found to increase with inflation step and was larger following negative feedback stimuli (bursts) as compared with positive feedback (successful inflations). These findings corroborate the functional involvement of the P2 in the early and preferential processing of stimuli with potential negative emotional consequences for the self.

We need to be aware however, that the ERP effects of inflation are confounded with the size of the bubble, which increased with every inflation step. Pfabigan, Sailer, and Lamm (2015) indicated that the amplitude of several ERP components, including the P2, is influenced by the physical size of feedback stimuli. The exact reason for the larger P2 to feedback stimuli of increasing size is unclear and could have various explanations (cf. Pfabigan et al., 2015) such as the differential expression of physical properties of large and small stimuli in the evoked potentials, the stronger salience and or potency of large stimuli to attract attention, and even the intrusive distance at which stimuli may be perceived. Note however that Kiat et al. (2016) contrasted inflating and deflating bubbles in the BART and found that in both conditions the P2 was found to increase with inflation/deflation step, suggesting that size of the gamble rather than the size of the bubble is what drives the P2 amplitude.

Most importantly for the purpose of the present experiment is that the P2 amplitude not only increased as a function of feedback valence and inflation step but it also varied as a function of the economic forecast. More specifically, P2 amplitudes were found to be larger, on average, when outcomes did not match the economic forecasts. That is, in the case of successful feedback, P2 amplitudes were larger when these outcomes were presented in the context of negative economic forecasts. Oppositely, for negative feedback stimuli, P2 amplitudes were larger when economic forecasts had been positive. These findings suggest that unexpectedly positive or negative outcome (considering the context of economic forecasting) generated a reward prediction error. Previous research investigating effects of predictive cueing in gambling paradigms has found similar reward prediction errors reflected in the amplitude of the P2 (Schaefer, Buratto, Goto, & Brotherhood, 2016), as well as other components, such as the FRN and the P3 (Hajcak, Holroyd, Moser, & Simons, 2005; Hajcak, Moser, Holroyd, & Simons, 2007; Mushtaq, Wilkie, Mon-Williams, & Schaefer, 2016; Walsh & Anderson, 2012; Wu & Zhou, 2009). The finding of a reward prediction error on the P2 in the current paradigm suggests that unexpected outcomes (i.e., not in accordance with the expectations formed on the basis of the forecasts) generated a stronger attentional orienting over and above the effects of stimulus frequency (i.e., bursts > successful inflations) and inflation step.

### N1

Over occipital regions, feedback stimuli generated an N1 component around 170 ms that increased in amplitude and decreased in latency as a function of inflation step. Previous studies have established that the visual N1 is sensitive to both exogenous factors, i.e., physical properties such as stimulus size and luminance (De Cesarei & Codispoti, 2006; Gannon, Knapp, Adams, Long, & Parks, 2016; Pfabigan et al., 2015; Wijers, Lange, Mulder, & Mulder, 1997) as well as endogenous factors such as selective attention in tasks requiring stimulus discrimination (Bradley, 2009; Hillyard, Vogel, & Luck, 1998). Accordingly, the effects of inflation step on the N1 could either reflect the size of feedback stimuli, which increased with inflation step, or increments in visual attention with the increasing size of gambles per inflation step (or a combination of the two). Two arguments from Kiat et al. (2016) support the latter attentional interpretation. In their ERP study of the BART, the increase in P2 amplitude with inflation/compression step suggests that feedback stimuli to sequential gambles in the BART increasingly recruited attentional resources. Furthermore, their results reveal an increase of the N1 amplitude with inflation step that is quite comparable with the effects on the P2 (see Kiat et al., 2016, Fig. 2,^2^). These findings suggest that the increase in N1 amplitude with inflation step in the current study, at least partly, reflect increased attentional processing of feedback stimuli with increasingly risky gambles in the BART (see Gu, Zhang, Luo, Wang, & Broster, 2018, for similar reasoning).

Interestingly, our analyses indicated that N1 amplitude was larger following negative feedback stimuli than in response to positive feedback, suggesting a negativity bias for attentive processing of negative emotional feedback over positive feedback. Although previous studies have reported a negativity bias for the P2 (see, e.g., Yuan et al., 2007), as far as we know, such an effect has not yet been reported for the N1. A possible account for the stronger N1 to bursts is that negative feedback stimuli triggered early recruitment of attentional resources (P2) (~130 ms), which was early enough to trigger enhancements in visual attention as reflected in the N1 (~170 ms). One possible reason for the early peak latency of the P2 is that the current paradigm used a clear and discriminative feature (i.e., colour) that allowed an early classification of the valence of the feedback stimulus. Future research could test this hypothesis by comparing the latency and amplitude of early ERP components to simple and complex feedback stimuli.

Importantly, negative economic forecasting increased the amplitude of the N1 to negative feedback, as compared with the positive economic forecasting condition. This effect corroborates the conclusion that economic forecasting changed participants’ mental model of the BART economy and their anticipation of negative feedback. Also, it supports our view that the effects in N1 amplitude reflect a measure of visual attention, which increases as a function of perceived risk. Interestingly, the effect of economic forecasting on the N1 was found selectively for negative feedback stimuli, and not for positive feedback stimuli (i.e., successful inflations). This finding suggests that participants’ anticipation of bubble burst was stronger in the negative forecasting condition than in the positive forecasting condition and selectively enhanced the processing of visual features associated with burst stimuli (cf. Müller & Keil, 2004).

### P3a

In line with other studies investigating electrophysiological responses in the BART, outcome stimuli generated a P3a component with a peak at around 270 ms for successive feedback stimuli, and more pronounced P3a with a somewhat later peak at around 340 ms following burst stimuli (Fein & Chang, 2008; Lannoy, D’Hondt, Dormal, Billieux, & Maurage, 2017). Previous research has found the P3a to be associated with involuntary orienting to unexpected salient stimuli in oddball paradigms (see review in Nieuwenhuis, De Geus, & Aston-Jones, 2011) and with the processing of outcomes following risky decisions in gambling paradigms (review in San Martín, 2012). Polich (2007) proposes that the P3a reflects the recruitment of a frontal attention mechanism that is triggered by incoming stimuli. In line with this idea, several papers have suggested that the frontal P2 and the P3a may involve the same component, with the P2 constituting an early phase of the P3a (Goyer et al., 2008; Polanía, Krajbich, Grueschow, & Ruff, 2014; Rigoni, Polezzi, Rumiati, Guarino, & Sartori, 2010; San Martín et al., 2013; San Martín, Kwak, Pearson, Woldorff, & Huettel, 2016), or, alternatively, that P2 and P3a are part of an oscillatory complex, caused by phase-resetting of theta (4–8 Hz) oscillations following stimulus onset (Foti, Weinberg, Dien, & Hajcak, 2011; Holroyd, Pakzad-Vaezi, & Krigolson, 2008; West et al., 2014).

ERP research in the domain of gambling has typically focused on brain potentials to outcome stimuli signaling success or failure. Studies have found P3a amplitude to vary as a function of valence and magnitude of the outcome (reviews in San Martín, 2012; West et al., 2014), with larger P3a amplitudes to negative outcomes than to positive outcomes, and to large gambles as compared with small gamble outcomes. In the current study, we found that P3a amplitude was larger for bubble bursts than to feedback of successful inflations. Furthermore, and in line with previous findings that P3a amplitude is sensitive to the magnitude of gambles, P3a amplitude in the present study was found to increase as a function of step size. In accordance with results on the P2 and the N1, these findings suggest that burst stimuli gathered more attention than successful inflations, and that outcomes of gambles with increasing magnitude received more attention.

Importantly, our analyses indicated that the amplitude of the P3a was not only modulated by valence and magnitude of the outcome stimuli but was also influenced by economic forecasting. More specifically, the increase in P3a amplitude with successive steps was found to be stronger following a negative forecasting messages than following positive economic forecast. This effect was found selectively in the ERPs to successful inflations, but not in response to bubble bursts. Considering the similarity in the effects of economic forecasting on the P2 and P3a to successful inflations (both set in after Step 4), and previous accounts that have argued for a functional overlap between these two components, it is likely that that the increase in P3a amplitude presents a similar reward prediction error (Fischer & Ullsperger, 2013; Ullsperger, Fischer, Nigbur, & Endrass, 2014) as in the case of the P2. In functional terms, this finding implies that unexpected positive outcomes in the context of negative economic forecasts caused a stronger orienting of attention to the outcome of the gamble.

### P3b

Following the P3a, a P3b developed over a centroparietal regions with a peak at around 410 ms for successful feedback stimuli. Comparable with the pattern of effects for the P3a, P3b amplitude was larger and peaked later (at around 570 ms) for bubble bursts than for successful inflations. Previous research has suggested that the P3b reflects the evaluation and encoding of novel and motivationally significant stimuli or events (reviews in Nieuwenhuis et al., 2004; Polich, 2007) that may inform future behavioural responses to the same stimuli or conditions. The P3b is found to be larger for stimuli with emotional valence than neutral stimuli (e.g., Delplanque et al., 2004; Delplanque, Silvert, Hot, & Sequeira, 2005; Keil et al., 2002), and for arousing stimuli than nonarousing stimuli (Rozenkrants & Polich, 2008). In gambling paradigms, P3b is larger in response to rewards than to losses (Hajcak et al., 2005; Hajcak et al., 2007) and is found to increase as a function of the absolute value or magnitude of gambles (e.g., Wu & Zhou, 2009; Yeung & Sanfey, 2004). Interestingly, the P3b has also been associated with adjustments of behaviour in task performance in subsequent trials (Chase et al., 2011; Fisher & Ullsperger, 2013; Gu et al., 2018; Mushtaq et al., 2016) and with the encoding of information in memory, supporting later recall and recognition (Donchin et al. 1984; Fabiani, Karis, & Donchin, 1990; Paller, Kutas, & Mayes, 1987; see a review in Wagner, Koutstaal, & Schachter, 1999). In the context of the present paradigm the P3b could reflect the encoding of trial outcomes to update the mental representation of the *p* distribution of bursts and successful inflations in the BART to optimize future financial decision-making.

Similar to the pattern of effects for the P2, N1, and P3a components, the P3b was found to be larger to bursts than to successful inflations, and analysis indicated that the P3b amplitude increased as a function of inflation step. These findings are in line with studies that have found the P3b amplitude to be influenced by the frequency of stimuli (review in Nieuwenhuis et al., 2004), and the magnitude of gambles (e.g., Wu & Zhou, 2009; Yeung and Sanfey, 2004). It is relevant to point out that the P3b is largely unaffected by physical properties of stimuli (Nieuwenhuis et al., 2004). Hence, differences in the size of stimuli per inflation step can be ruled out as a confounding factor for the P3b. Most importantly, however, the amplitude of the P3b to successful inflations was modulated by the valence of the economic forecast. More specifically, the increase in P3b amplitude with successive steps was larger following negative economic forecasts than following positive forecasts. This finding suggests that the reward prediction error to successful inflations following negative economic predictions also influenced the P3b component. This finding implies stronger engagement of different cognitive or affective functions such as attention, reward, arousal, and memory encoding (Nieuwenhuis et al., 2004; Pfabigan, Alexopoulos, Bauer, & Sailer, 2011; Polich, 2007; San Martín, 2012) in response to unexpected positive outcomes in the BART.

Altogether, the findings on the four consecutive ERP components present a consistent picture. In all components we found that inflation step parametrically enhanced the amplitude of all four ERP components. These effects probably reflect stronger attentional engagement to stimuli of greater importance (Goyer et al., 2008; Kiat et al., 2016; San Martín, 2012; West et al., 2014; Wu & Zhou, 2009; Yeung and Sanfey, 2004). In addition, and most importantly considering the goal of the present study, the amplitudes of all four components were influenced by economic forecasting. In cases of the P2, P3a, and P3b, the effects of economic forecasting probably reflect a reward prediction error (Fischer & Ullsperger, 2013; Hajcak et al., 2005; Hajcak et al., 2007; Mushtaq et al., 2016; Schaefer et al., 2016; Ullsperger et al., 2014; Walsh & Anderson, 2012; Wu & Zhou, 2009) that captures attention when the outcomes in the BART are unexpected in light of the preceding economic forecast. The similarity in ERP effects across the four components suggests a close functional coupling between the different processing stages of outcome stimuli in the BART. These findings match previous accounts that have stressed functional relations between the P2, P3a, and P3b. More specifically, it has been suggested that the consecutive P2, FRN, and P3a reflect a frontal theta oscillatory response supporting attentional orienting to presented stimuli (West et al., 2014). According to Polich (2007) and recent work by Bachman and Bernat (2018), attentional orienting as reflected in theta may also contribute to the P3b.

The current findings provide support for the idea that the predictive processing framework may be well suited to explain how beliefs about the economy may influence people’s economic choices. Participants’ electrophysiological responses to positive and negative outcomes indicate that economic forecasting messages influenced participants’ predictions about the outcomes of their risky decisions. This finding is consistent with predictive processing models in the domain of action (Friston, 2010; Pickering & Clark, 2014; Ridderinkhof & Brass, 2015), which suggest that people form specific top-down expectations about the outcomes of their actions that serve as a perceptual filter in the bottom-up processing of action consequences (Lin et al., 2012; Melloni, Schwiedrzik, Müller, Rodriguez, & Singer, 2011). Analysis of risk taking over time indicated that participants did not update their false beliefs about the BART economy, which points to the idea that false beliefs higher up in the perceptual hierarchy may be quite difficult to change, especially when the available evidence is noisy or ambiguous (Petropoulos Petalas et al., 2017). Similarly, the state of the general economy may be hard to estimate, given the complex economic indices, and individuals may be selectively processing information that is consistent with their view (Kaaronen, 2018). In addition to predictive processing, our findings may also be compatible with model-based reinforcement learning, which suggests that animals and humans may instantaneously change their reward-based decision-making depending on the (economical) context (Lee, Seo, & Jung, 2012; Trueblood, Brown, Heathcote, & Busemeyer, 2013; Zhang et al., 2013). The current paradigm may provide a fitting case wherein the integration between reinforcement learning and predictive processing (cf. Alexander & Brown, 2018) may be investigated in future studies.

### Concluding remarks

Contemporary interest from multiple scientific domains focuses on the influence of economic forecasting on financial decision-making. In this paper, we have discussed behavioural and ERP findings from an economic decision-making experiment to investigate the hypothesis that positive and negative economic forecasts can influence individual’s internal model of the economic reality and influence their subsequent financial decision-making. In accordance with this idea, economic forecasts were found to influence individuals’ risk taking and their ERPs to decision outcomes, whereby unexpected outcomes were processed more attentively than outcomes that were in line with previous economic forecasts. These findings confirm the self-fulfilling prophecy effect in economic forecasting and corroborate existing models that have suggested a causal relationship between economic news and economic decision-making at a macro level.

## Electronic supplementary material

ESM 1 (PDF 1072 kb)

## Footnotes

1 Previous studies investigating outcome processing in decision making have also identified reward prediction errors to be reflected in the FRN (Bai, Katahira, & Ohira, 2015; Fein & Chang, 2008; Yi Huang & Yu, 2014; San Martín, 2012; note that the FRN has also been labeled fERN, MFN, and FN; see Proudfit, 2015). A few EEG studies have run varying versions of the BART, among which some have reported an FRN observed at frontocentral electrode sites approximately 240 ms following balloon bursts (e.g., Fein & Chang, 2008; Takács et al., 2015), while others have failed to identify it (Gu et al., 2018). In our study, we found no indications of an FRN. A possible reason for this may be that a strong reward-related positivity (RewP) component with a similar frontocentral topography, such as the P3a in the present study, masked the FRN (cf. Proudfit, 2015).

2 Note, though, that a statistical analysis of this effect is not reported in the Kiat et al. (2016) paper.
