# Supplementary material for: Event-related brain potentials reflect predictive coding of anticipated economic change
Source: Cogn Affect Behav Neurosci. 2020 Aug 18;20(5):961–82. doi: 10.3758/s13415-020-00813-5 (PMC7497516; doi:10.3758/s13415-020-00813-5)
Supplement: Supplementary file 1 — (PDF 1072 kb) [file 13415_2020_813_MOESM1_ESM.pdf]

Supplementary Material

Table S3

Bubble values associated with burst probabilities in successive inflation steps.

Inflation step number	bubble value	*p* of bursting
1	0.00	0
2	0.10	.08
3	0.20	.16
4	0.35	.25
5	0.50	.33
6	0.70	.41
7	0.95	.50
8	1.25	.58
9	1.40	.66
10	1.90	.75
11	2.45	.83
12	3.10	.91
13	3.90	1

Note. Risk taking was quantified as mean number of inflations performed across all trials in the BART. In each trial, participants could inflate the bubble at least two times and maximally 12 times.

Table S4

Information on number of epochs used for ERP identification and analysis

Inflation step number	Successful inflations			Bubble bursts		
	participants (*n*)	epochs (*M*)	(*SD*)	participants (*n*)	epochs (*M*)	(*SD*)
1	20	1224,3	105,4	-	-	-
2	20	1138,3	60,2	20	108,3	17,2
3	20	1019,0	65,1	20	104,7	15,9
4	20	885,0	55,1	20	96,3	2,9
5	20	739,0	74,5	20	86,7	11,7
6	20	509,0	93,3	20	73,7	17,9
7	19	341,0	93,7	18	59,0	9,8
8	18	195,3	73,6	17	37,0	16,1
9	10	80,7	43,8	13	13,7	15,2
10	7	36,3	24,1	10	9,0	7,8
11	0	11,7	10,7	0	0	0
12	0	2,0	3,5	0	0	0

Note. The number of epochs (*M* and *SD*) used for identifying EPR components was collapsed across experimental blocks and participants. Some participants (*n*) were more risk averse and gave fewer responses resulting in less epochs/trials for higher inflation steps. The reduced signal to noise ratio at these higher inflation steps is taken into account by the mixed model analysis (see paper) that assigns statistical weight of condition cells as a function of the number of observations

For Review Only

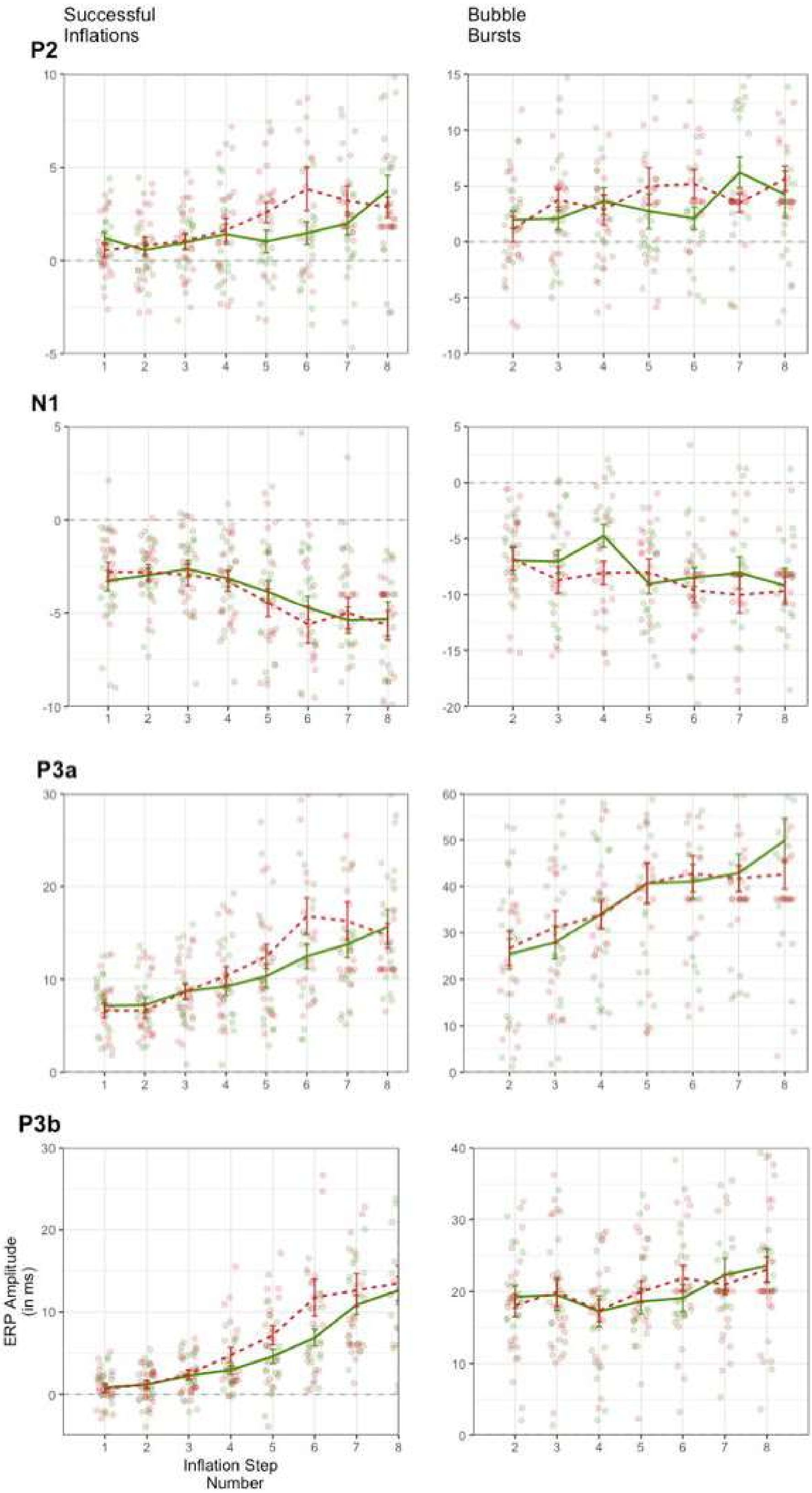

Figure S7. Summary of the P2, N1, P3a and P3b ERP components' mean amplitudes as a function of inflation step number 1–8. Solid green lines correspond to positive and dashed red lines correspond to negative economic forecasting; red and green dots

indicate individual participant data points. Whiskers indicate mean standard error.

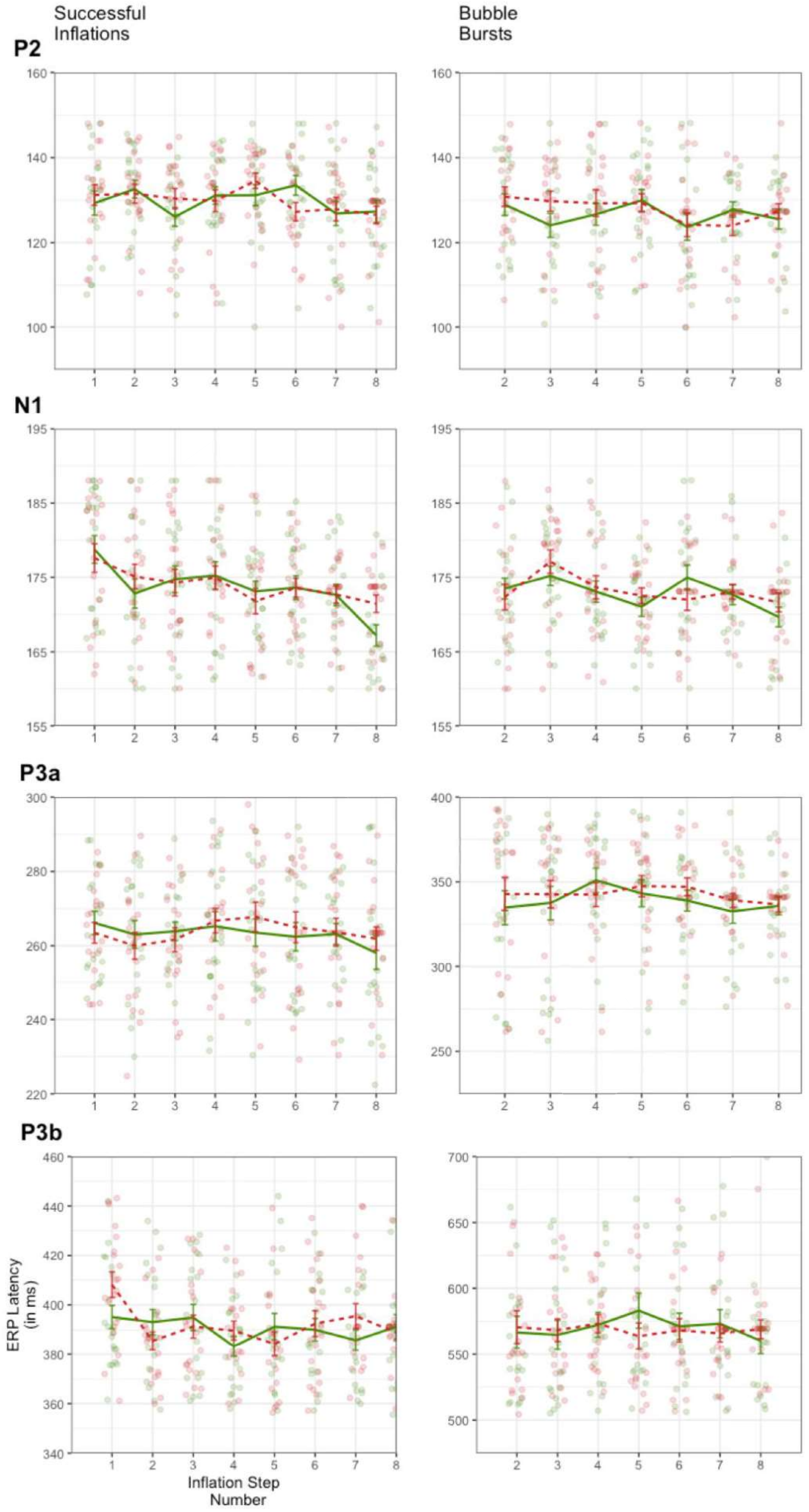

Figure S8. Summary of the P2, N1, P3a and P3b ERP components' mean latencies as a function of inflation step number 1–8. Solid green lines correspond to positive and dashed red lines correspond to negative economic forecasting; red and green dots

indicate individual participant data points. Whiskers indicate mean standard error.

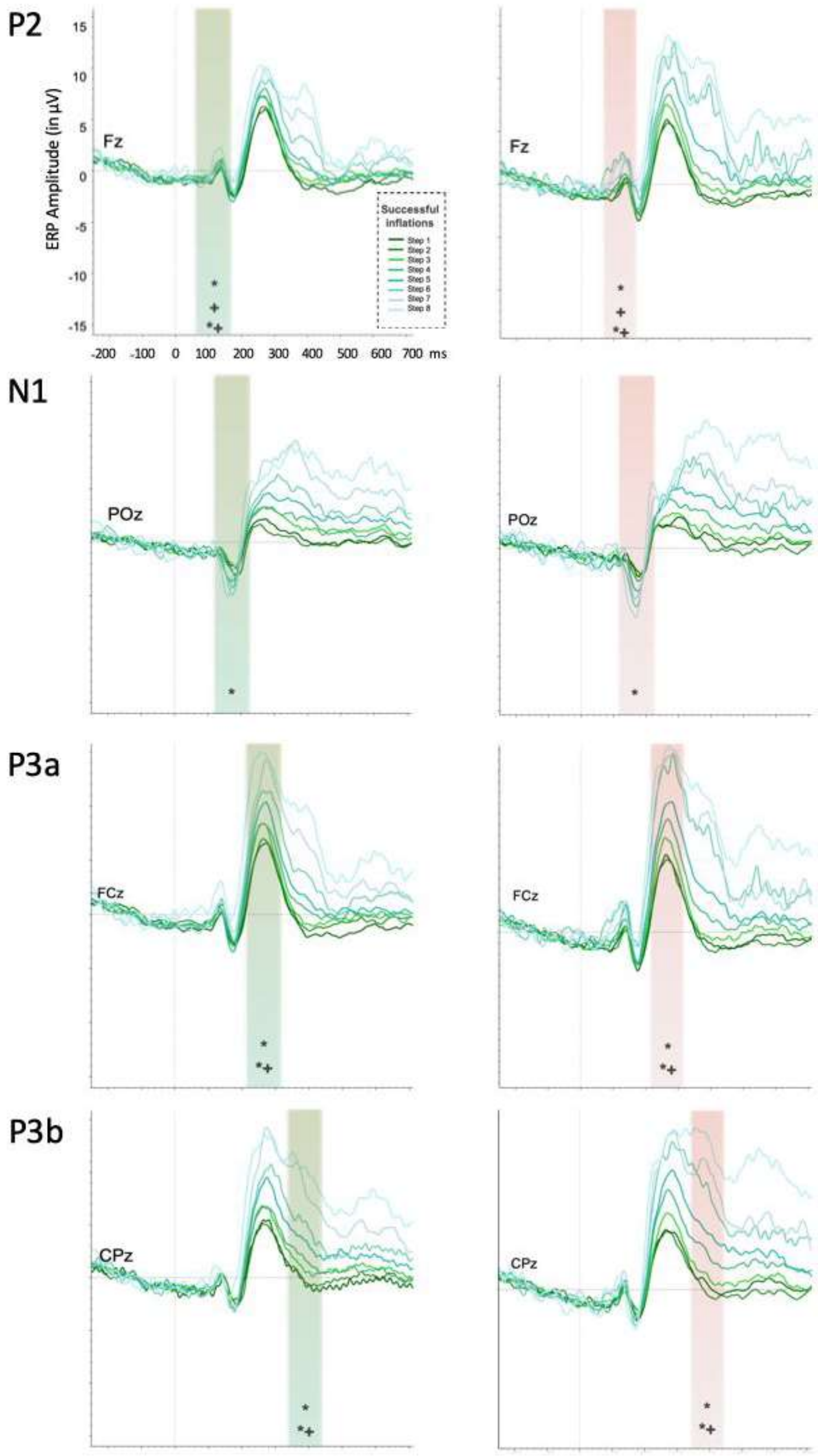

Figure S9: ERPs per inflation step number 1–8 time-locked to successful bubble inflation feedback screens at peaking electrode sites for the positive and for the negative economic forecasting conditions. Shaded regions (green for positive and red for negative forecasting) mark the time range of interest corresponding to each ERP component. Single asterisks indicate significant differences for the main effect of Step; the plus

symbol indicates significant differences for the main effect of Condition; the combination of an asterisk and a plus symbol is used for denoting an interaction effect of Step x Condition.

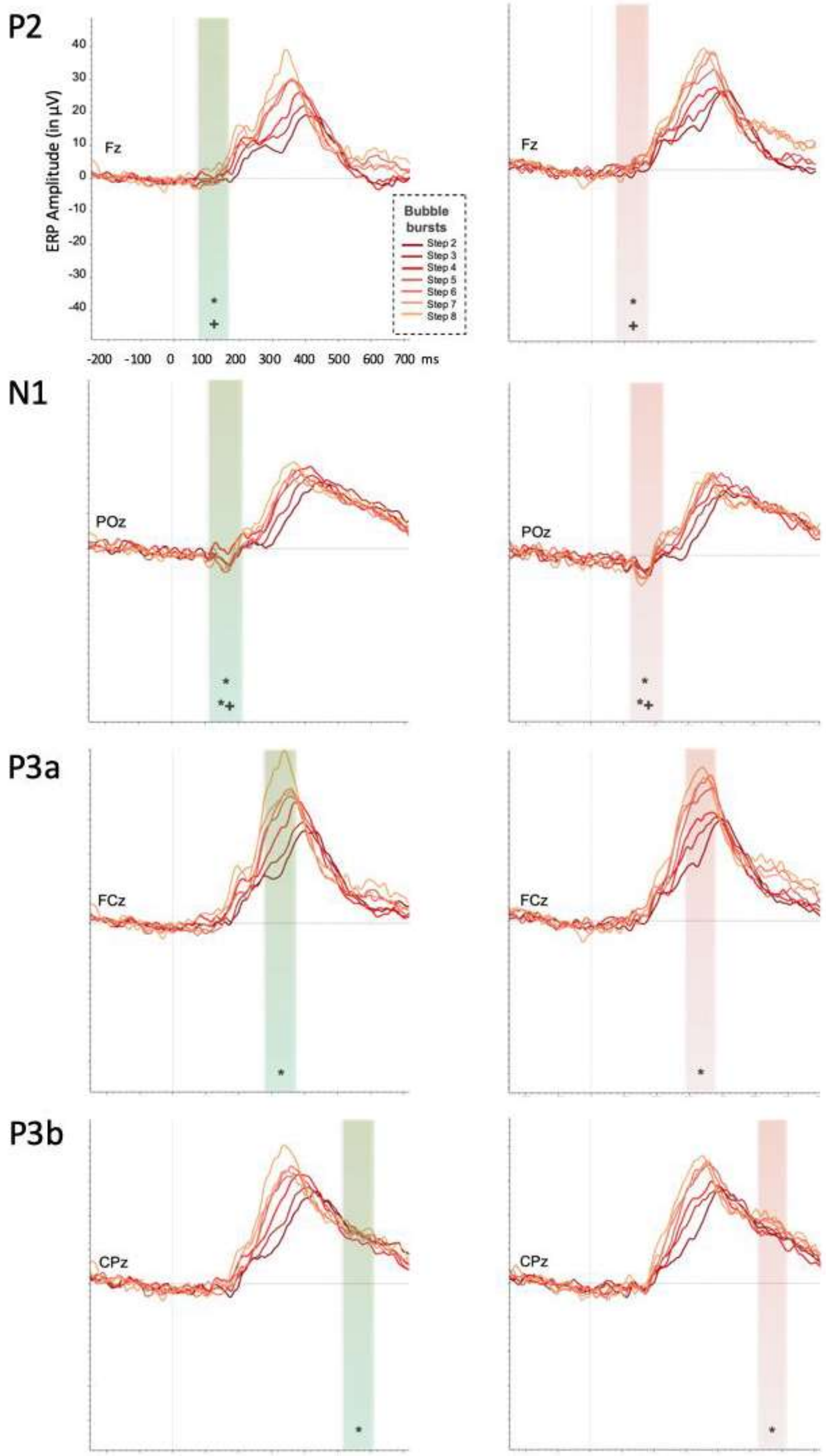

Figure S10: ERPs per inflation step number 2–8 time-locked to bubble bursts at peaking electrode sites, for the positive and negative economic forecasting conditions. Shaded regions (green for positive and red for negative forecasting) mark the time range of interest corresponding to each ERP component. Single asterisks indicate significant differences for the main effect

of Step; the plus symbol indicates significant differences for the main effect of Condition; the combination of an asterisk and a plus symbol is used for denoting an interaction effect of Step x Condition.

For Review Only
